# Inverse relation of body weight with short-term and long-term mortality following hip fracture surgery: a meta-analysis

**DOI:** 10.1186/s13018-022-03131-3

**Published:** 2022-04-26

**Authors:** Tzu-I Yang, Yu-Hang Chen, Ming-Hsiu Chiang, Yi-Jie Kuo, Yu-Pin Chen

**Affiliations:** 1 Department of General Medicine, Changhua Christian Hospital, Changhua, Taiwan; 2 Department of General Medicine, Kaohsiung Chang Gung Memorial Hospital, Kaohsiung, Taiwan; 3 Department of Orthopedics, Wan Fang Hospital, Taipei Medical University, No. 111, Sec. 3, Xinglong Rd., Wenshan Dist., Taipei City, 116 Taiwan; 4 Department of Orthopedics, School of Medicine, College of Medicine, Taipei Medical University, Taipei, Taiwan

**Keywords:** Body weight, Obesity, Surgery, Mortality

## Abstract

**Background:**

The obesity paradox, which suggests that high body weight is positively associated with survival in some diseases, has not been proven in patients with hip fracture. In this study, meta-analysis of previous studies on the impacts of body weight on postoperative mortality following hip fracture surgery in older adults was conducted.

**Methods:**

PubMed, Embase, and Cochrane library were searched for studies investigating the correlation between mortality after hip fracture surgery and body weight. The search main items included: (“Body mass index” OR “BMI” or “body weight”) and (“hip fracture” or “hip fractures”). Studies contained data on short-term (≤ 30-day) and long-term (≥ 1 year) mortality after hip fracture and its association with distinct body weight or BMI groups were reported as full-text articles were included in this meta-analysis.

**Results:**

Eleven separate studies were included. The definitions of underweight and obesity differed among the included studies, but the majority of the enrolled studies used the average body weight definition of a BMI of 18.5 to 24.9 kg/m^2^; underweight referred to a BMI of < 18.5 kg/m^2^; and obesity pertained to a BMI of > 30 kg/m^2^. Based on the generalized definitions of body-weight groups from the enrolled studies, the group with obesity had lower long-term (odds ratio [OR]: 0.63, 95% CI: 0.50–0.79, *P* < 0.00001) and short-term (OR: 0.63, 95% CI: 0.58–0.68, *P* ≤ 0.00001) mortality rates after hip fracture surgery when compared with patients with average-weight group. However, compared with the average-weight group, the underweight group had higher long-term (OR: 1.51, 95% CI: 1.15-1.98, *P*=0.003) and short-term (OR: 1.49, 95% CI: 1.29-1.72, *P*<0.00001) mortality rates after hip fracture surgery.

**Conclusions:**

Current evidence demonstrates an inverse relation of body weight with long-term and short-term mortality after hip fracture surgery in older adults.

**Supplementary Information:**

The online version contains supplementary material available at 10.1186/s13018-022-03131-3.

## Background

Hip fractures, which are associated with high morbidity, mortality, and high health-care costs, are a global challenge [1]. The reported in-hospital mortality rate for older patients receiving hip fracture surgery was estimated to be approximately 2% [2], and the 1-year mortality rate following hip fracture surgery ranges from 14 to 18.1% [3]. In addition, one-third of the older population is reportedly severely dependent at 1 year after hip fracture surgery [4]. Moreover, the incidence of hip fracture appears to be increasing especially in developing populations in Asia [5]. With the increasing number of hip fractures as populations age [6], the considerable socioeconomic impact of hip fractures is expected to continue to increase in the near future. Hence, adopting a stratified care approach that accounts for personalized risks to reduce mortality after hip fracture in older patients is indispensable.

Obesity is among the principal causes of morbidity and mortality. Patients with clinical obesity are predisposed to several diseases including diabetes mellitus, hypertension, stroke, and coronary artery disease [7]. Obesity is also considered an independent predictor of survival and complication rates following major surgical procedures [8]. Evidence also indicates that obesity increases the complexity of joint replacement surgery and is associated with high risks of complications following arthroplasty including periprosthetic infection, wound dehiscence, and prosthesis dislocation in the general adult population [9]. Low body weight may be another key predictor of poor health outcomes following major surgery. A meta-analysis revealed that following coronary artery bypass grafting, patients with underweight had higher risk of mortality than the general adult patients with overweight [10]. A longitudinal follow-up study on hip joint replacement also revealed that general older patients (over the age of 65) with underweight (not with obesity) had significantly greater mortality risk than patients with average body weight [11], which highlights the controversy over the effect of body mass index (BMI) on mortality following major orthopedic surgery.

Studies have reported a phenomenon called the obesity paradox, which suggests that high body weight is positively associated with old age survival in the case of some chronic diseases and following hip fracture surgery [12]. However, the association between BMI and survival after hip fracture remains unclear in the literature. Because of the lack of strong evidence on this topic, this is the first meta-analysis of previous studies investigating the impact of body weight on postoperative mortality following hip fracture surgery.

## Methods

### Study design and search strategy

This meta-analysis was conducted in accordance with the Preferred Reporting Items for Systematic Review and Meta-Analysis (PRISMA) guidelines (Additional file 1: Table S1) [13]. PubMed, Embase, and Cochrane library databases were searched for articles reporting on the association between body weight and mortality after hip fracture that were published from September 1, 2011, through September 30, 2021. The search strategy involved the following set of keywords: (“Body mass index” OR “BMI” or “body weight”) and (“hip fracture” or “hip fractures”). No language restrictions were imposed, and the reference lists of the included studies were screened. Titles and abstracts were screened to identify relevant studies. Citations were retrieved as full text for more detailed evaluation of applicability and Endnote was used as a tool to assist screening. Doctors who were specialized in hip fracture surgery or in geriatric patient care were invited to review the screening process to identify any additional studies.

Initially, citations were identified which included patients with hip fracture and were evaluated with BMI or morality. Two reviewers (YHC and YTI) were independently incorporated to screen the initial search results for potentially eligible studies. The reviewers resolved disagreements by consensus, and if consensus was not achieved, a third reviewer will be incorporated to make a decision. All identified studies were then retrieved in full text, as were any studies where the abstract was unavailable or where ambiguity existed. To be included, studies were required to include patients that had hip fracture, grouping based on BMI, include mortality as one of the outcomes, and be published in English. There was a publication year limits of 2005. This study is registered on the International Prospective Register of Systematic Reviews (CRD42021261629).

### Eligibility criteria

The criteria for study inclusion were as follows: the study (i) contained data on patient mortality after hip fracture and its association with body weight or BMI, (ii) reported in full-text format, (iii) had follow-up times with reported results on postoperative mortality, and (iv) were conducted after the World Health Organization (WHO)’s definition of obesity as BMI > 30 kg/m^2^ (2005 WHO Surveillance of Risk Factors report).

The study exclusion criteria were as follows: the study (i) had a population of size < 100 (to prevent sampling errors common in meta-analyses with small sample sizes), (ii) involved patients with multiple fractures, (iii) had no body weight or BMI data, (iv) had no mortality data, (v) did not group result based on different category of BMI, (vi) a meta-analysis, systematic review, or not experimental study, and (vii) fully text not available.

### Data extraction and management

Two review authors (Y.-H.C. and T.-I.Y.) independently extracted data including study timeframe, publication year, country, design, setting, number of patients, body-weight groups, mean age, sex distribution, follow-up duration, and mortality from the individual studies. The extracted information was checked by a third author (Y.-P.C.).

Data for body-weight groups were extracted for meta-analysis based on the definitions of underweight, average weight, and obesity in each enrolled study. However, considering the variations on the definitions for body-weight groups among studies, we performed subgroup analysis from studies with uniformed definitions for body-weight groups based on the World Health Organization (WHO) classification: underweight (BMI < 18.5 kg/m^2^), average weight (BMI 18.5–24.9 kg/m^2^), and obesity (BMI ≥ 30 kg/m^2^).

The methodological quality of each study was also assessed and scored independently by the 2 main review authors in accordance with the Newcastle–Ottawa Scale (NOS) [14]. In case of disagreement between the 2 review authors, the third author intervened to achieve a resolution and make a final decision. The NOS was used to assess each study on the basis of 8 items categorized into 3 categories: (i) the selection of the study groups, (ii) comparability of the groups, (iii) and how diet patterns are ascertained (objectively or subjectively). Stars are awarded for each quality item, and the highest-quality studies are awarded 9 stars. A study is considered to be of good quality if rated with 3 or 4 stars in the selection domain, 1 or 2 stars in comparability domain, and 2 or 3 stars in the outcome or exposure domain.

### Outcome measurements

The primary outcome of interest was the long-term (≥ 1-year) and short-term (≤ 30-day) mortality rates in the group with obesity versus average-weight group after hip fracture. The secondary outcome of interest was the long-term and short-term mortality rates in the underweight versus average-weight group. Hazard ratios for each outcome were also calculated in this meta-analysis.

### Statistical analysis and data synthesis

In this meta-analysis study, the extracted data were collated using ReviewManager 5.3 [15].

The mortality rates of the average weight, underweight, and group with obesity were calculated using the odds ratios (ORs) and 95% CIs from each included study. Hazard ratios were also collected. Statistical significance was defined as *P* < 0.05. Heterogeneity among the included studies was determined using the Chi-squared, Cochrane *Q*, and *I*^2^ tests. A Cochrane *Q* result with *P* < 0.1 and *I*^2^ > 50% was considered representative of significant heterogeneity [16]. A random-effects model was selected on the basis of the included studies’ heterogeneity. If 2 separate populations were discussed in the same paper and no overlap was present, 2 separate groups of data were considered. Possible publication biases were visually assessed using a funnel plot that represented most included studies. Egger’s test was used to identify small-study effects; *P* < 0.05 suggested a small-study effect.

For a forest plot with > 5 studies, publication bias was assessed using a symmetric funnel plot.

## Result

### Identification of included studies

**Fig. 1:**
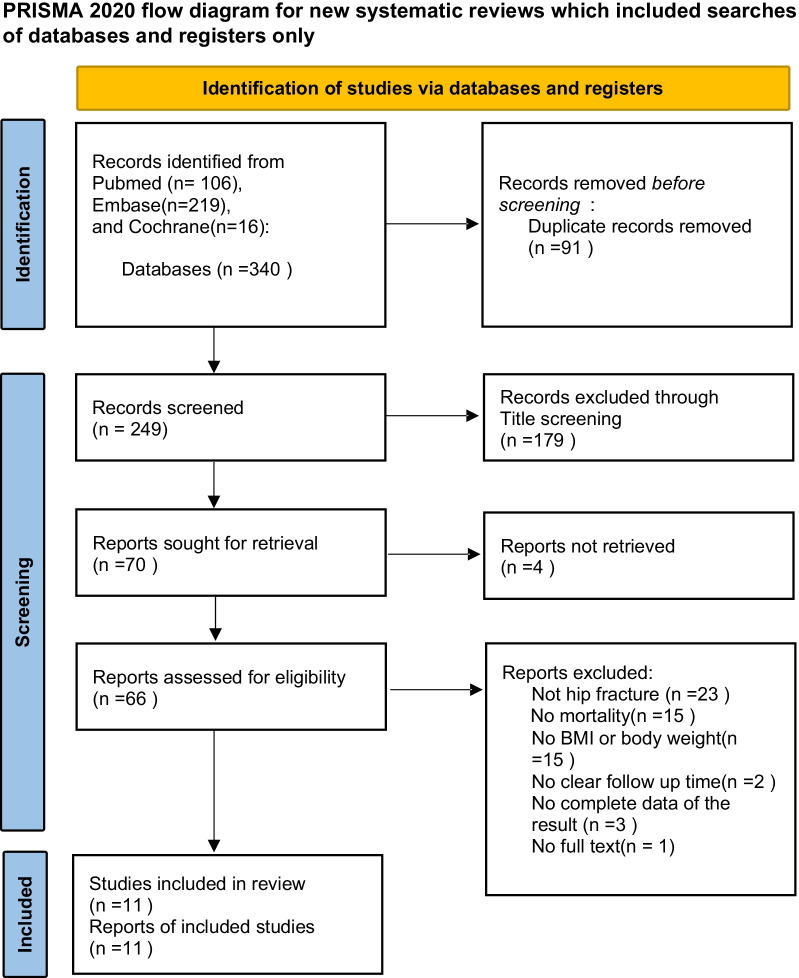
Flowchart of study selection

Through database searches, 340 articles were retrieved from the 3 databases (PubMed, Cochrane Library, and Embase). A total of 11 articles include 13 separate study datasets. (2 datasets were extracted from a 2016 study by Pederson [17], and 2 datasets were extracted from a 2020 study by Tahir [18].) Definitions of body-weight groups differed among the included studies. Underweight group was defined by BMI of ≤ 18.5 kg/m^2^ in 7 articles, BMI of ≤ 20 kg/m^2^ in 2 articles, and BMI of ≤ 22 kg/m^2^ in 2 articles. Obesity group was defined by BMI of ≥ 25 kg/m^2^ in one article, BMI of ≥ 26 kg/m^2^ in one article, and BMI of ≥ 30 kg/m^2^ in 9 articles. The excluded studies (and reasons for exclusion) are detailed in Additional file 2: Table S2. The selection process is presented as a PRISMA flow diagram in Fig. 1 [19].

### Quality assessment of included studies

The results of NOS-based methodological analysis of the included studies are presented in Additional file 3: Table S3. Among the included studies, 3 were graded as high quality (≥ 7 points) and 4 were graded as moderate quality (≥ 4 and < 7 points).

### Study characteristics

The characteristics of the included studies are detailed in Table 1. The 11 studies consisted of both prospective and retrospective trials and enrolled a total of 56,380 patients. The average age of the population ranged from 60 to 90 years. Female patients were predominant, accounting for 69% of all the included participants. The definitions of underweight, average body weight, and obesity varied slightly among the studies. The majority of the enrolled studies used the average body weight definition of a BMI of 18.5 to 24.9 kg/m^2^; underweight referred to a BMI of < 18.5 kg/m^2^; and obesity pertained to a BMI of > 30 kg/m^2^.

**Table 1:** Characteristics of included studies

Authors	Year	Country	Study design	Total (% female)	Mean age	Underweight definition Decease/total number (%)	Average weight definition Decease/total number (%)	Obesity/definition Decease/total number (%)	Mortality Follow-up	Hazard ratio provided
Chaudhry et al. [24]	2021	USA	Retrospective studies	104,468 (69%)	NA	BMI < 18.5	BMI: 18.5–24.9	BMI > 30	30 days	No
						856/9292 (9.2%)	3421/49,916 (6.9%)	708/16,153 (4.8%)		
Müller et al. [26]	2020	Munich, Germany	Retrospective single-center cohort study	950 (67.1%)	74.8 ± 13.51	BMI < 18.5	BMI: 18.5–25	BMI > 30	< 30 days	No
						2/80 (2.5%)	17/570 (3%)	1/59 (1.7%)		
Pedersen et al. (transfuse) (non-transfused) [17]	2016	Denmark	Prospective population-based study	10,527	NA	BMI < 18.5	BMI: 18.5–25	BMI > 30	1 year	Yes
						No data/5010	No data/25,639	No data/2889		
Prieto‐Alhambra et al. [22]	2014	Spain	Retrospective population- based cohort study	6988 (71.3%)	79.3 ± 11.8	BMI < 18.5	BMI: 18.5–25	BMI > 30	1.17 (0.53–2.02) year	Yes
						26/79 (32.9%)	308/1353 (22.8%)	187/1315 (14.2%)		
Solbakken et al. [21]	2017	Norway	Matched cohort study	3177 (65%)	80.6	BMI < 18.5	BMI: 18.5–25	BMI > 30	2–9 years	Yes
						59/76 (78%)	888/1372 (65%)	291/476 (61%)		
Kirkland et al. [25]	2011	Minnesota, USA	Retrospective cohort chart review	485 (73%)	64–90	BMI < 18.5	BMI: 18.5–25	BMI > 30	30 days	No, only odds ratio
						8/42 (19%)	19/241 (7.9%)	1/67 (1.5%)		
Kimura et al. [28]	2019	Kyushu, Japan	Retrospective study	497 (78.5%)	80.1 ± 7.9	BMI < 18.5	BMI: 18.5–25	BMI > = 25	1 year	Yes
						26/158 (16.5%)	17/295 (5.7%)	2/44 (4.5%)		
Tahir et al. [18]	2020	Karachi, Pakistan	Prospective study	490 (68.6%)	NA	BMI < 20	BMI: 20–25	BMI > 30		
						25/73 (34.2%)	40/146 (25.9)	17/117 (14.5%)	1 years	No
						2/73 (2.7%)	2/146 (1.3%)	0/117 (0%)	30 days	No
Akinleye et al. [23]	2018	USA	Prospective study	15,108 (62%)	69.8–77	BMI < 20	BMI: 20–29	BMI > 30	30 days	No
						196/2414 (8.12%)	552/10,734 (5.14%)	74/1960 (3.8%)		
Modig et al. [12]	2019	Sweden	Prospective cohort Study	17,756 (68.3%)	82.7 ± 8.0	BMI < 22	BMI: 22–25	BMI > 30	1 year	Yes
						1778/5986 (29.7%)	1253/5494 (22.8%)	222/1503 (14.8%)		
Flodin et al. [27]	2016	Stockholm, Sweden	Prospective multicenter study	843 (73%)	82 ± 7	BMI < 22	BMI: 22–26	BMI > 26	1 year	No
						58/364 (16%)	61/341 (18%)	9/138 (6%)		

*BMI* body mass index

### Effect of obesity on mortality after hip fracture surgery

**Fig. 2:**
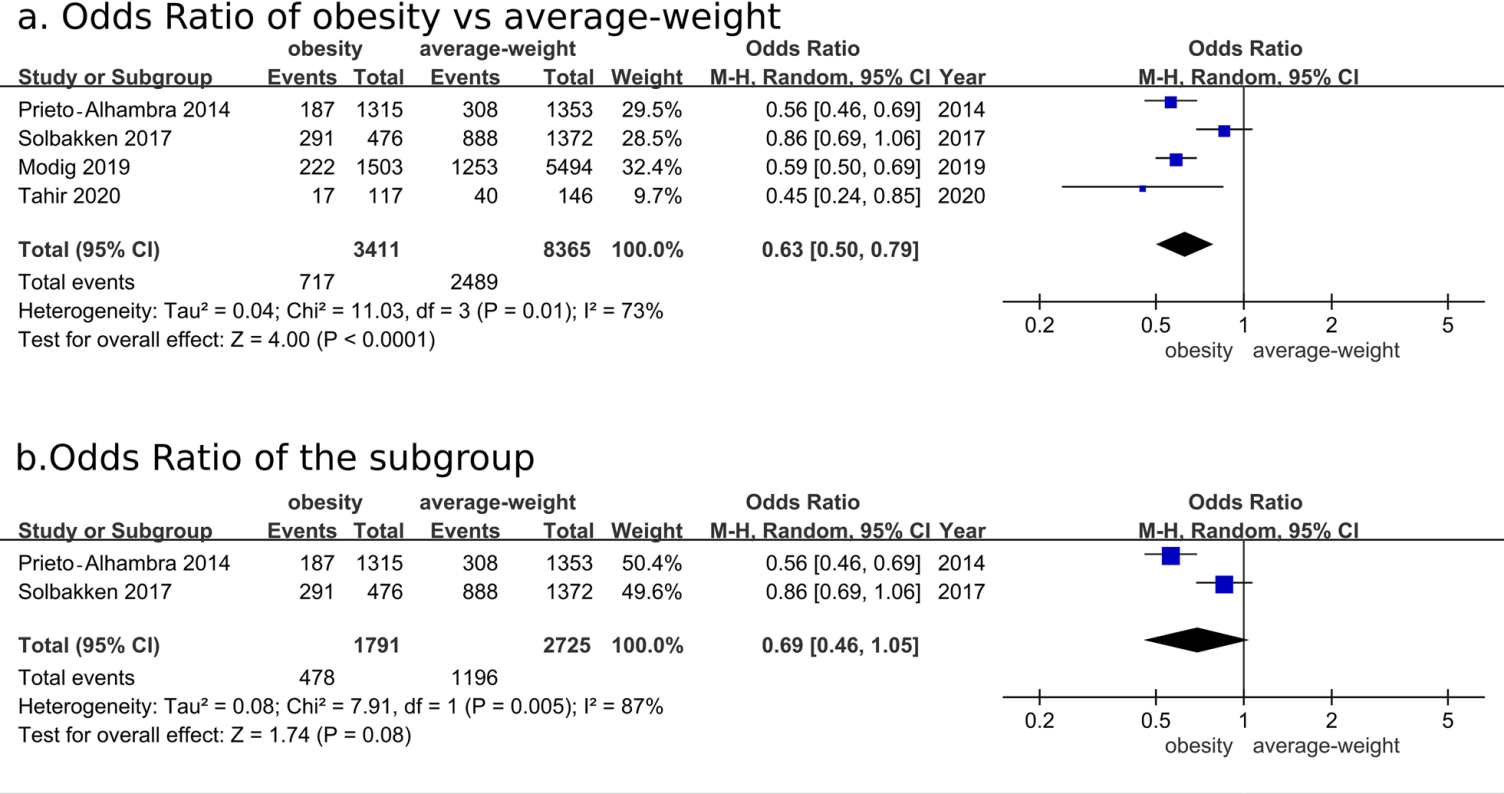
Comparison of long-term mortality rate between the group with obesity and average-weight groups after hip fracture surgery. **a** Overall analysis for odds ratio, **b** subgroup analysis using uniformed definitions of obesity (BMI ≥ 30 kg/m^2^) and average weight (BMI of 18.5–24.9 kg/m^2^)

Four eligible studies were included in the meta-analysis of the long-term mortality risk after hip fracture surgery. Figure 2a indicates an OR of 0.63 (95% CI: 0.50–0.79; *P* < 0.00001) for the risk of long-term mortality after hip fracture surgery in the group with obesity compared with the average-weight group [12, 18, 20, 21]. Three studies were included in the meta-analysis to reveal a higher pooled hazard ratio of 0.66 (95% CI: 0.59–0.73; *P* < 0.01) for long-term mortality in the group with obesity compared with the average-weight group in Additional file 4: Fig. S1 [17, 21, 22]. In addition, a subgroup analysis for 2 studies with uniformed definitions of obesity (BMI ≥ 30 kg/m^2^) and average weight (BMI of 18.5–24.9 kg/m^2^) indicates an OR of 0.69 (0.46, 1.05; *P* = 0.08) (Fig. 2b) [21, 22].

**Fig. 3:**
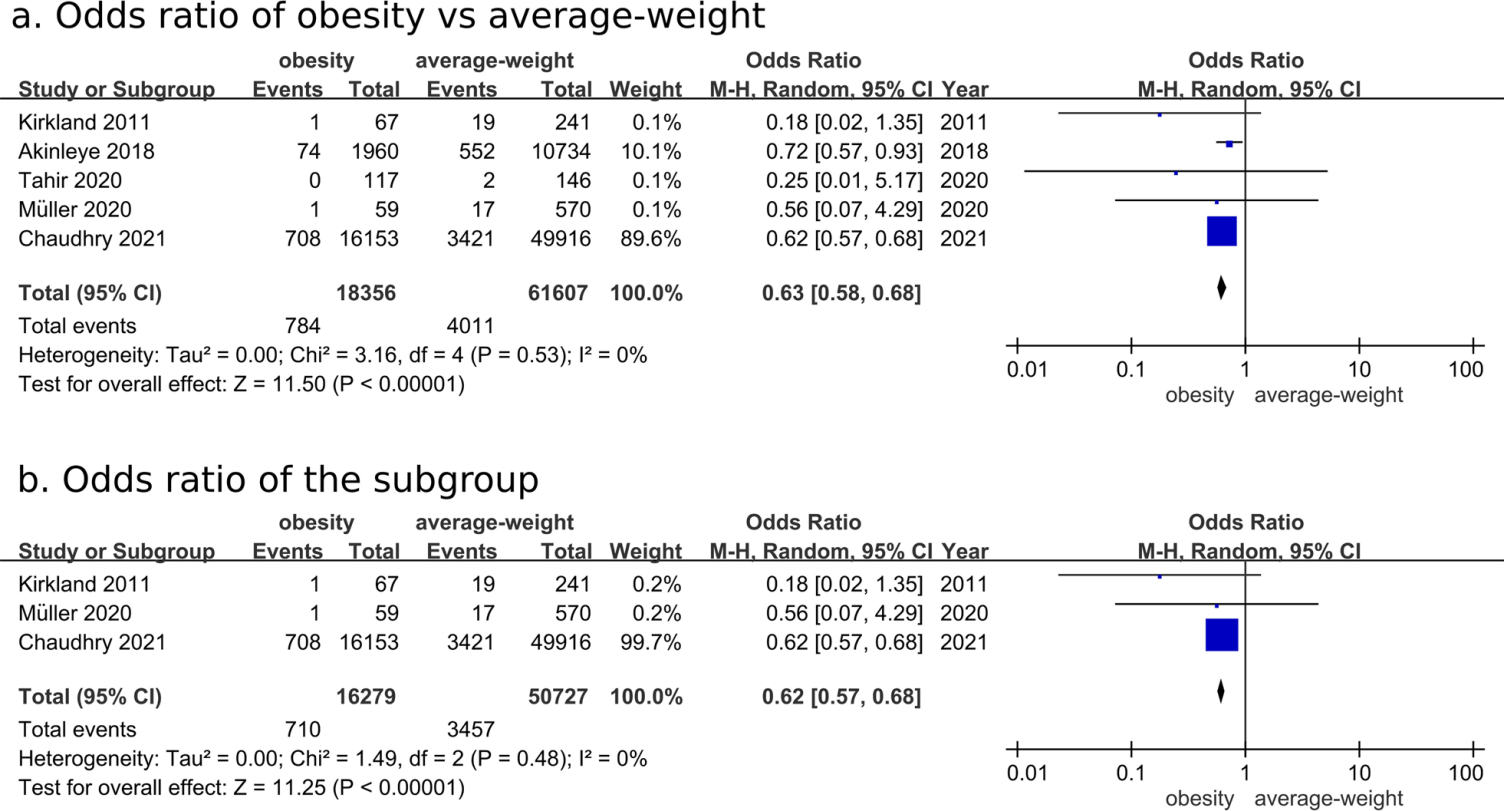
Comparison of short-term mortality rate between the group with obesity and average-weight group after hip fracture surgery. **a** Overall analysis for odds ratio, **b** subgroup analysis using uniformed definitions of obesity (BMI ≥ 30 kg/m^2^) and average weight (BMI of 18.5–24.9 kg/m^2^)

Five studies were meta-analyzed for the risk of short-term (≤ 30-day) mortality after hip fracture surgery [18, 23–26]. The OR was 0.63 (95% CI: 0.58–0.68; *P* < 0.00001) for short-term mortality in the group with obesity compared with the average-weight group (Fig. 3a). Subgroup analysis including 3 studies with uniformed definitions of obesity (BMI ≥ 30 kg/m^2^) and average weight (BMI of 18.5–24.9 kg/m^2^) showed consistent OR result of 0.62 (95% CI; 0.57–0.68; *P* < 0.00001) (Fig. 3b) [24–26].

### Effect of underweight on mortality after hip fracture surgery

**Fig. 4:**
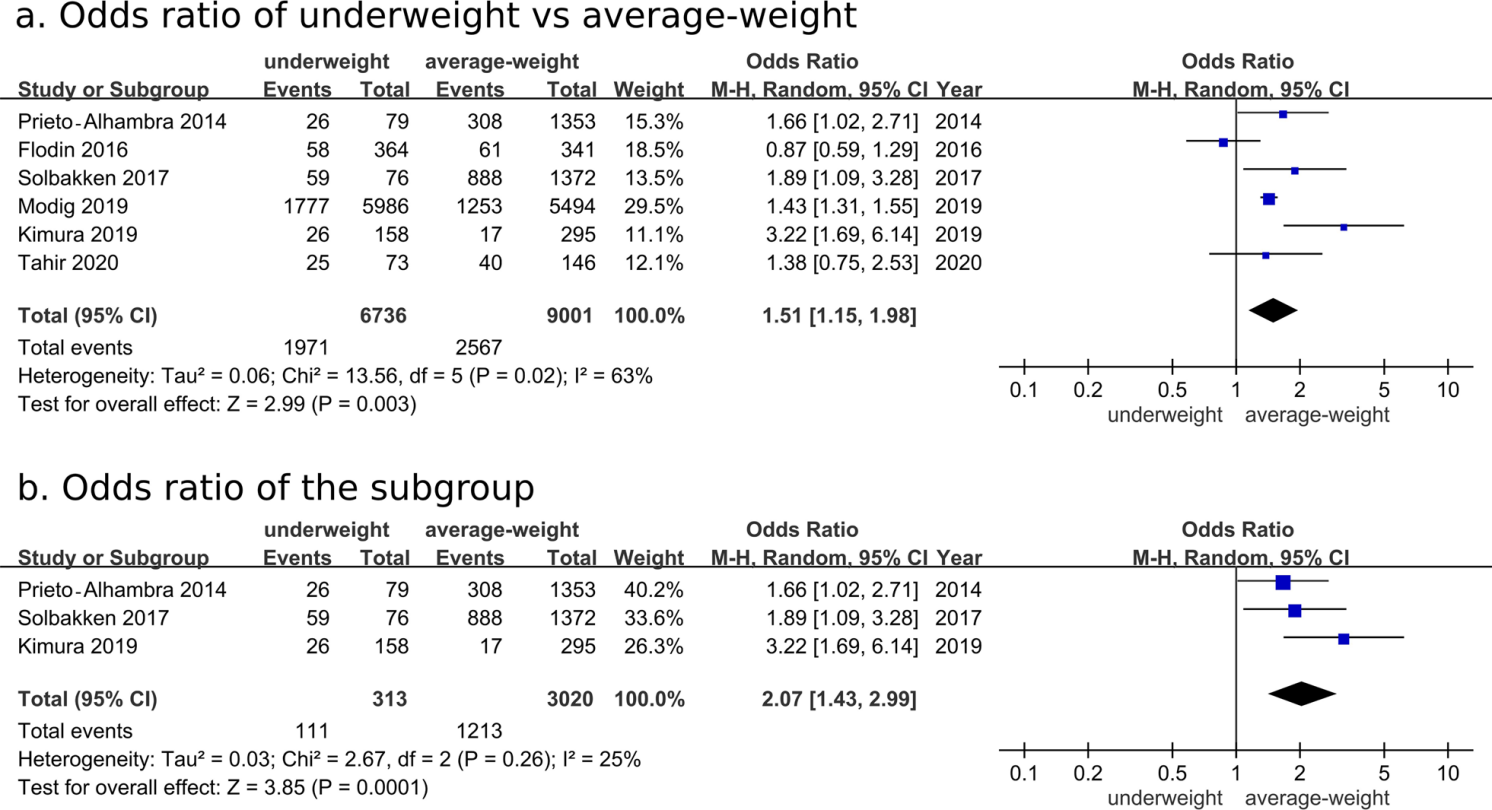
Comparison of long-term mortality rate between average-weight and underweight groups after hip fracture surgery. **a** Overall analysis for odds ratio, **b** subgroup analysis using uniformed definitions of underweight (BMI < 18.5 kg/m^2^) and average weight (BMI of 18.5–24.9 kg/m^2^)

Six studies were included for meta-analysis of the long-term mortality after hip fracture surgery [12, 18, 21, 22, 27, 28]. Compared with the average-weight group, the underweight group had a significantly higher risk of long-term mortality (OR: 1.51, 95% CI: 1.15–1.98, *P* = 0.003; Fig. 4a). The meta-analysis of 4 studies also revealed a higher pooled hazard ratio (1.47, 95% CI: 1.33–1.62, *P* ≤ 0.01) for long-term mortality in the underweight group than in average-weight group after hip fracture in Additional file 4: Fig. S1 [12, 17, 22, 28]. Subgroup analysis including **3** studies with uniformed definitions of underweight (BMI < 18.5 kg/m^2^) and average weight (BMI of 18.5–24.9 kg/m^2^) showed an OR of 2.07 (95% CI: 1.43–2.99; *P* = 0.0001) (Fig. 4b) [21, 22, 28].

**Fig. 5:**
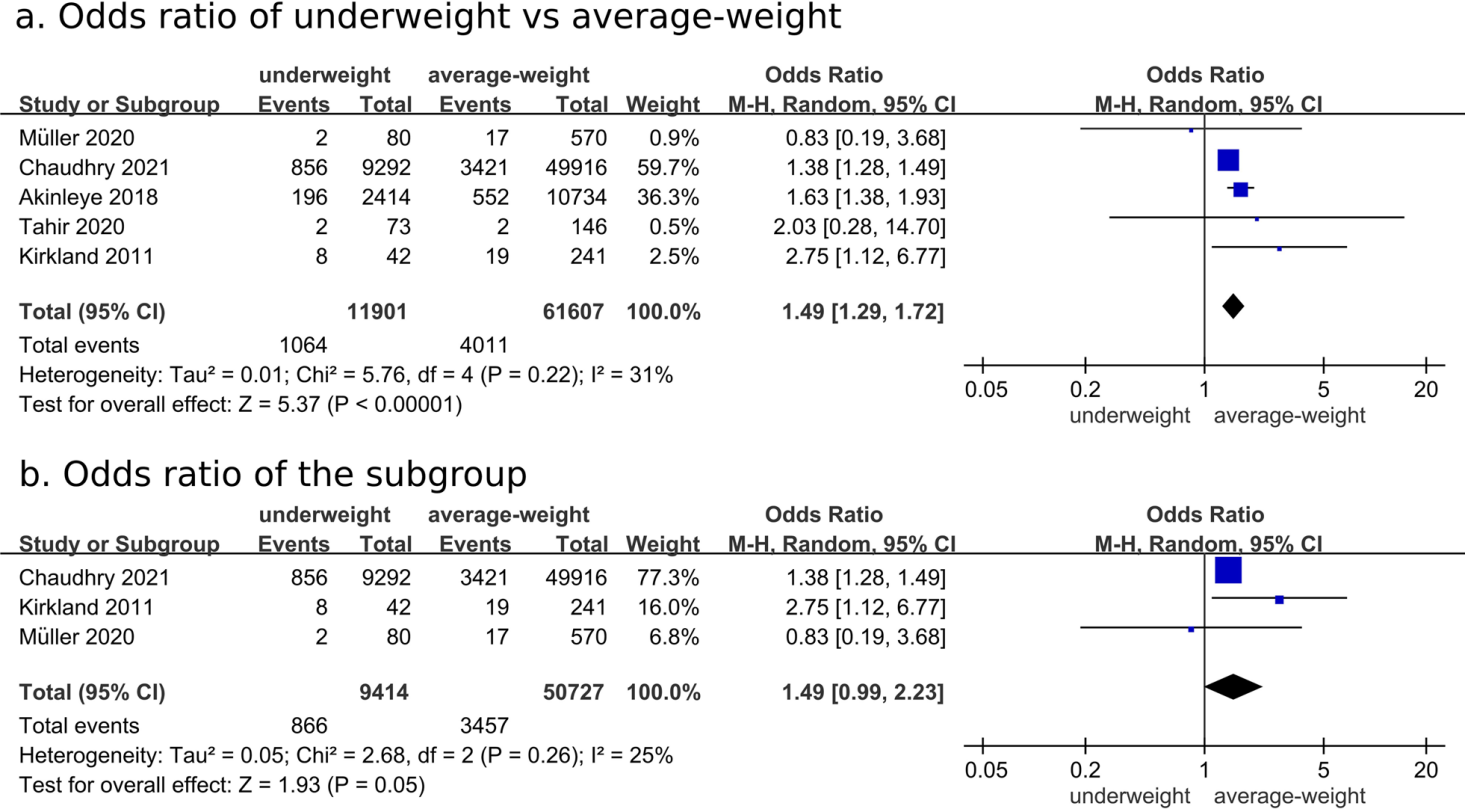
Comparison of short-term mortality rate between average-weight and underweight groups after hip fracture surgery. **a** Overall analysis for odds ratio, **b** subgroup analysis using uniformed definitions of underweight (BMI < 18.5 kg/m^2^) and average weight (BMI of 18.5–24.9 kg/m^2^)

**Fig. 6:**
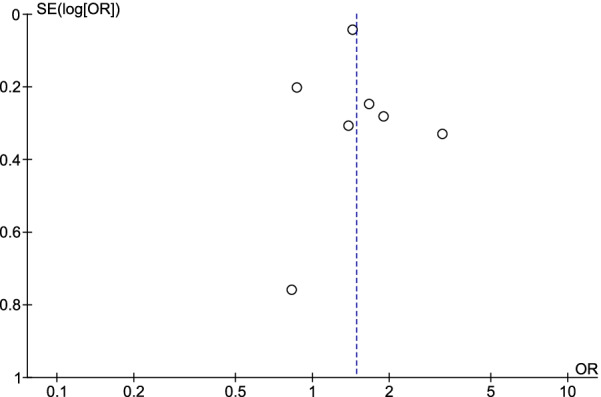
Funnel plot of comparison: average-weight versus underweight groups after hip fracture surgery, outcome: long-term mortality

Five studies were meta-analyzed for the risk of short-term (≤ 30-day) mortality after hip fracture surgery [18, 23–26]. The OR for short-term mortality in the underweight group was 1.49 (95% CI: 1.29–1.72, *P* < 0.00001) in comparison with the average-weight group (Fig. 5a), which adds to the results on the higher risk of long-term mortality following hip fracture surgery. Subgroup analysis of 3 studies with uniformed definitions of underweight (BMI < 18.5 kg/m^2^) and average weight (BMI of 18.5–24.9 kg/m^2^) disclosed OR of 1.49 (95% CI: 0.99–2.23, *P* = 0.05) (Fig. 5b) [24–26]. A symmetric funnel plot for long-term mortality provides evidence of publication bias in the included studies (Fig. 6).

## Discussion

In this meta-analysis study, based on the generalized definitions of body-weight groups from enrolled studies, individuals with obesity were discovered to have lower risks of short-term and long-term mortality after hip fracture surgery compared with patients with average-weight group. By contrast, underweight was associated with higher risks of short-term and long-term mortality after hip fracture surgery when compared with average weight. However, although with consistent trends on the clinical relationship between body-weight groups and postoperative mortality, subgroup analysis for studies with restrict definitions of underweight (BMI < 18.5 kg/m^2^), average weight (BMI of 18.5–24.9 kg/m^2^), and obesity (BMI ≥ 30 kg/m^2^) only revealed the significant protective effect of obesity on the short-term mortality and detrimental effect of underweight on the long-term mortality after hip fracture surgery when compared with average weight.

Hip fractures are associated with increased mortality in older adults [2]. Although hip fractures are not a direct cause of death, comorbidities resulting from hip fracture and subsequent immobility play a critical role in the development of thrombosis-related complications [29]. Coronary artery disease, cancer, and stroke are reportedly the leading causes of death in patients with hip fracture [30]. Obesity is a well-known independent predictor of cardiovascular diseases even in the absence of other risk factors in the general adulthood [31] and must be inferred to be a predictor of adverse cardiovascular events and associated mortality following hip fracture surgery in older adults. However, other evidence notably indicates that obesity can be a protective factor that promotes survival after the onset of cardiovascular diseases [32]. This phenomenon of inverse relation of body weight with postoperative mortality has been called the obesity paradox [12]. In a meta-analysis, Orepoulous et al. reported lower all-cause mortality in the general adults patients with overweight and obesity in the event of heart failure [33]. In addition, the recent study has reported that obesity is associated with superior survival outcomes in specific diseases, including stroke [34]. Our meta-analysis also demonstrates the obesity paradox in patients with hip fracture, indicating the protective effect of obesity for survival after hip fracture surgery.

The physiology underlying the obesity paradox is not well understood. Some explanations describe the impact of body weight on mortality after hip fracture surgery. After a hip fracture, patients may suffer a series of stress reactions promoted by the inflammatory response and remain in a hypercatabolic status for up to 3 months [35]. The large amount of adipose tissue in patients with obesity can act as an energy reserve, resulting in more favorable neuroendocrine profiles to meet the increased postoperative metabolic demands. Therefore, patients with obesity may have greater ability to tolerating stress responses after hip fracture [36]. Adipose tissue benefits from enrichment of tumor necrosis factor α receptors, which can induce a less prothrombotic state and neutralize substances associated with deleterious metabolic effects [37]. In addition, patients with obesity have been observed to have lower levels of natriuretic peptide, a key substance for lipolysis that affects the rate of peptide degradation due to the adipose tissue-induced increase in clearance receptors [38]. Reduced amounts of natriuretic peptides in patients with obesity may also reinforce protection against endotoxin-related and cytokine-related inflammation [37]. Although patients with hip fracture have relatively low bone mineral density, which is reportedly associated with higher mortality risk [12], high body weight can counteract age-related bone loss. However, the excess weight may weaken the protective effect and become detrimental to bone health in patients with obesity [39].

In this meta-analysis, underweight was notably associated with a higher rate of mortality after hip fracture surgery than was average weight; this finding is consistent with findings from several studies suggesting that older patients with underweight have increased incidence of postoperative complications and higher mortality rates [40]. In older adults, being underweight is associated with malnutrition and osteoporosis [41] and may also be accompanied by sarcopenia [42]. Osteosarcopenia, which is a new geriatric syndrome involving the coexistence of osteoporosis and sarcopenia, is defined as the pathological loss of bone density and skeletal muscle mass. The combined effect of these two chronic musculoskeletal conditions is severe and may result in frailty in older adults [43]. Older patients with hip fractures along with frailty and malnutrition have poor response to stress, functional recovery, and wound healing after hip fracture surgery [44]. In addition, osteosarcopenia is regarded as a negative predictor of functional outcomes and mortality in older patients following hip fracture surgery [45]. One study also revealed that compared with average weight, underweight is typically associated with poorer immune response and lower respiratory muscle and peripheral skeletal muscle strength, which lead to elevated risk of respiratory disease–associated mortality [46]. These pathological changes in patients with underweight may explain this meta-analysis’ finding of the detrimental effect of underweight on mortality following hip fracture surgery in older adults.

This systematic review study has several limitations. First, the study focused on all-cause mortality (rather than cause-specific mortality). Therefore, we could not definitively explain the relationship between body weight and specific causes of mortality following hip fracture surgery. Second, body weight was only classified using BMI in this study. Although BMI is a common clinical measure for obesity, it cannot fully reflect detailed information on body composition such as muscle and skeletal mass, which may affect metabolic reserves, recovery of function, and mortality outcomes. Moreover, the BMI data in the included studies were only registered at baseline; weight change was not monitored. Finally, the definitions of underweight and obesity differed among the included studies, which may have affected the reported postoperative mortality rates. Recent studies proposed the optimal geriatric BMI defined as ranging from 23 to 29.9 kg/m^2^ in adults ≥ 65 years of age [47, 48]. However, the majority of the enrolled studies used the definitions based on the WHO classification: underweight (BMI < 18.5 kg/m^2^), average weight (BMI 18.5–24.9 kg/m^2^), and obesity (BMI ≥ 30 kg/m^2^).

Thus, we further performed subgroup analysis that grouped the patients based on the WHO classification in order to eliminate the potential bias from the varied definitions for body-weight groups among enrolled studies. However, despite these limitations, this is the first meta-analysis to provide strong evidence regarding the impacts of body weight on postoperative mortality following hip fracture surgery and can serve as a valuable reference for clinicians promoting a stratified care approach in which the risk of mortality is reduced after hip fracture in older patients with underweight in particular.

## Conclusion

Current evidence demonstrates the inverse relation of body weight with short-term and long-term mortality following hip fracture surgery in older adults. Clinicians must focus on minimizing the influence of underweight status on postoperative mortality in this vulnerable population. However, owing to the definitions of body-weight groups differing among studies, further research with a larger sample and restrict definitions of body-weight groups is essential to verify the clinical relationship between body-weight groups and postoperative mortality in older patients with hip fracture.

## Supplementary Information

**Additional file 1.** Prisma checklist.

**Additional file 2.** The reasons for exclusion of excluded studies.

**Additional file 3.** Quality assessment of the included studies on NOS-based methodological analysis.

**Additional file 4.** Comparison of long-term mortality rate in hazard ratios after hip fracture surgery (a) underweight vs average-weight groups (b) obesity vs average weight group.

## Data Availability

All data generated or analyzed during this study are included in these published article [12, 17, 18, 21–28].

## Abbreviations

BMI: Body mass index
WHO: World Health Organization
NOS: Newcastle–Ottawa Scale
ORs: Odds ratios

## References

[1] Lesic A, Jarebinski M, Pekmezovic T (2007). Epidemiology of hip fractures in Belgrade, Serbia Montenegro, 1990–2000. Arch Orthop Trauma Surg.

[2] Chiang MH, Lee HJ, Kuo YJ (2021). Predictors of in-hospital mortality in older adults undergoing hip fracture surgery: a case-control study. Geriatr Orthop Surg Rehabil.

[3] Lee TC, Ho PS, Lin HT (2017). One-year readmission risk and mortality after hip fracture surgery: a national population-based study in Taiwan. Aging Dis.

[4] Chen Y-P, Kuo Y-J, Liu C-H (2021). Prognostic factors for 1-year functional outcome, quality of life, care demands, and mortality after surgery in Taiwanese geriatric patients with a hip fracture: a prospective cohort study. Ther Adv Musculoskelet Dis..

[5] Curtis EM, Moon RJ, Harvey NC (2017). The impact of fragility fracture and approaches to osteoporosis risk assessment worldwide. Bone.

[6] Cheung C-L, Ang SB, Chadha M (2018). An updated hip fracture projection in Asia: the Asian Federation of Osteoporosis Societies study. Osteoporos Sarcopenia.

[7] Haslam DW, James WP (2005). Obesity. Lancet.

[8] Turrentine FE, Hanks JB, Schirmer BD (2012). The relationship between body mass index and 30-day mortality risk, by principal surgical procedure. Arch Surg.

[9] Luger M, Hochgatterer R, Schopper C (2021). Obesity in short stem total hip arthroplasty using a minimally invasive supine anterolateral approach—a risk factor for short-term complications?. Int Orthop.

[10] Protopapas AD (2016). Does body mass index affect mortality in coronary surgery?. Open Cardiovasc Med J.

[11] Woo SH, Cha DH, Park EC (2019). The association of under-weight and obesity with mortality after hip arthroplasty. Age Ageing.

[12] Modig K, Erdefelt A, Mellner C (2019). "Obesity paradox" holds true for patients with hip fracture: a registry-based cohort study. J Bone Jt Surg Am.

[13] Moher D, Liberati A, Tetzlaff J (2009). Preferred reporting items for systematic reviews and meta-analyses: the PRISMA statement. PLOS Med.

[14] Wells GA, Wells G, Shea B (2014). The Newcastle–Ottawa Scale (NOS) for assessing the quality of nonrandomised studies in meta-analyses.

[15] 2014. Review Manager 5 (RevMan 5). Copenhagen: Nordic Cochrane Centre, The Cochrane Collaboration.

[16] Thorlund K, Imberger G, Johnston BC (2012). Evolution of heterogeneity (I2) estimates and their 95% confidence intervals in large meta-analyses. PLoS ONE.

[17] Pedersen AB, Cronin Fenton D, Nørgaard M (2016). Body mass index, risk of allogeneic red blood cell transfusion, and mortality in elderly patients undergoing hip fracture surgery. Osteoporos Int.

[18] Tahir M, Ahmed N, Samejo MQA (2020). The phenomenon of "obesity paradox" in neck of femur fractures. Pak J Med Sci.

[19] Moher D, Liberati A, Tetzlaff J (2009). Preferred reporting items for systematic reviews and meta-analyses: the PRISMA statement. BMJ.

[20] Salvador Marín J, Ferrández Martínez FJ, Fuster Such C (2021). Risk factors for high length of hospital stay and in-hospital mortality in hip fractures in the elderly. Rev Esp Cir Ortop Traumatol (Engl Ed)..

[21] Solbakken SM, Meyer HE, Stigum H (2017). Excess mortality following hip fracture: impact of self-perceived health, smoking, and body mass index. A NOREPOS study. Osteoporos Int.

[22] Prieto-Alhambra D, Premaor MO, Avilés FF (2014). Relationship between mortality and BMI after fracture: a population-based study of men and women aged ≥ 40 years. J Bone Miner Res.

[23] Akinleye SD, Garofolo G, Culbertson MD (2018). The role of BMI in hip fracture surgery. Geriatr Orthop Surg Rehabil.

[24] Chaudhry YP, Rao SS, Puvanesarajah V (2021). Complications and 30-day mortality rate after hip fracture surgery in superobese patients. J Orthop Trauma.

[25] Kirkland LL, Kashiwagi DT, Burton MC (2011). The Charlson Comorbidity Index Score as a predictor of 30-day mortality after hip fracture surgery. Am J Med Qual.

[26] Müller M, Gutwerk A, Greve F (2020). The association between high body mass index and early clinical outcomes in patients with proximal femur fractures. J Clin Med.

[27] Flodin L, Laurin A, Lökk J (2016). Increased 1-year survival and discharge to independent living in overweight hip fracture patients: a prospective study of 843 patients. Acta Orthop.

[28] Kimura A, Matsumoto Y, Wakata Y (2019). Predictive factors of mortality of patients with fragility hip fractures at 1 year after discharge: a multicenter, retrospective study in the northern Kyushu district of Japan. J Orthop Surg (Hong Kong).

[29] Yuan X, Zhu Z, Yao J (2019). Epidemiology and risk factors for deep vein thrombosis in patients with hip fractures. Int J Clin Exp Med.

[30] Cauley JA (2013). Public health impact of osteoporosis. J Gerontol A Biol Sci Med Sci.

[31] Khan SS, Ning H, Wilkins JT (2018). Association of body mass index with lifetime risk of cardiovascular disease and compression of morbidity. JAMA Cardiol.

[32] Carbone S, Canada JM, Billingsley HE (2019). Obesity paradox in cardiovascular disease: where do we stand?. Vasc Health Risk Manag.

[33] Oreopoulos A, McAlister FA, Kalantar-Zadeh K (2009). The relationship between body mass index, treatment, and mortality in patients with established coronary artery disease: a report from APPROACH. Eur Heart J.

[34] Wassertheil-Smoller S, Fann C, Allman RM (2000). Relation of low body mass to death and stroke in the systolic hypertension in the elderly program. The SHEP Cooperative Research Group. Arch Intern Med.

[35] Hedström M, Ljungqvist O, Cederholm T (2006). Metabolism and catabolism in hip fracture patients: nutritional and anabolic intervention—a review. Acta Orthop.

[36] Hébuterne X, Bermon S, Schneider SM (2001). Ageing and muscle: the effects of malnutrition, re-nutrition, and physical exercise. Curr Opin Clin Nutr Metab Care.

[37] Mohamed-Ali V, Goodrick S, Bulmer K (1999). Production of soluble tumor necrosis factor receptors by human subcutaneous adipose tissue in vivo. Am J Physiol.

[38] Sengenès C, Berlan M, De Glisezinski I (2000). Natriuretic peptides: a new lipolytic pathway in human adipocytes. Faseb j.

[39] Maïmoun L, Mura T, Leprieur E (2016). Impact of obesity on bone mass throughout adult life: influence of gender and severity of obesity. Bone.

[40] Traven SA, Reeves RA, Althoff AD (2019). New five-factor modified frailty index predicts morbidity and mortality in geriatric hip fractures. J Orthop Trauma.

[41] Coin A, Sergi G, Benincà P (2000). Bone mineral density and body composition in underweight and normal elderly subjects. Osteoporos Int.

[42] Tonial PC, Colussi EL, Alves ALS (2020). Prevalence of sarcopenia in elderly users of the primary health care system. Nutr Hosp.

[43] Paintin J, Cooper C, Dennison E (2018). Osteosarcopenia. Br J Hosp Med (Lond).

[44] Meesters DM, Wijnands KAP, Brink PRG (2018). Malnutrition and fracture healing: are specific deficiencies in amino acids important in nonunion development?. Nutrients.

[45] Chiang MH, Kuo YJ, Chen YP (2021). The association between sarcopenia and postoperative outcomes among older adults with hip fracture: a systematic review. J Appl Gerontol.

[46] Engelen MP, Schols AM, Baken WC (1994). Nutritional depletion in relation to respiratory and peripheral skeletal muscle function in out-patients with COPD. Eur Respir J.

[47] Porter Starr KN, Bales CW (2015). Excessive body weight in older adults. Clin Geriatr Med.

[48] Winter JE, MacInnis RJ, Wattanapenpaiboon N (2014). BMI and all-cause mortality in older adults: a meta-analysis. Am J Clin Nutr.

