# Supplementary material for: Inverse relation of body weight with short-term and long-term mortality following hip fracture surgery: a meta-analysis
Source: J Orthop Surg Res. 2022 Apr 26;17:249. doi: 10.1186/s13018-022-03131-3 (PMC9044716; doi:10.1186/s13018-022-03131-3)
Supplement: Supplementary file 2 — Additional file 2. The reasons for exclusion of excluded studies. [file 13018_2022_3131_MOESM2_ESM.docx]

**Table S2: Excluded studies and reasons**

| **Reasons for exclusion:** | **Number** | **Reference** |
| --- | --- | --- |
| Studies that did not include participants with hip fracture through screening | 68 | (1-68) |
| Studies that were not BMI or body weight related through screening | 102 | (69-167) |
| Studies that were not retrievable through screening | 4 | (168-171) |
| Studies that were meta-analysis, systemic review, or case reports | 10 | (172-181) |
| Studies that did not provided precise fracture data | 23 | (182-204) |
| Studies that did not provided precise BMI or body weight grouping | 15 | (205-219) |
| Studies that did not provided mortality data | 15 | (123, 220-233) |
| Studies that did not provided complete follow up | 1 | (234) |
| Studies that did not provided complete data | 3 | (235-237) |
| Studies that was unavailable to full text | 1 | (238) |

Reference

1. Acharya KKV, Pandey V, Rao PS. Male osteoporosis: Our experience with an understudied disease. *Osteoporosis International* 2012;**23:** S687-S688.

2. Ahmed T, Steward J, O'Mahony MS. Esterases, frailty and mortality in older people in the community. *Age Ageing* 2011;**40:** ii2.

3. Arain SR, Riaz A, Nazir L, Umer TP, Rasool T. LOW BONE MINERAL DENSITY AMONG PATIENTS WITH NEWLY DIAGNOSED RHEUMATOID ARTHRITIS. *J Ayub Med Coll Abbottabad* 2016;**28:** 175-178.

4. Aubry-Rozier B, Stoll D, Krieg MA, Lamy O, Hans D. What was your fracture risk evaluated by FRAX® the day before your osteoporotic fracture? *Clin Rheumatol* 2013;**32:** 219-223.

5. Bakeri H, Luthra P. Management of a complex patient with anorexia nervosa, osteoporosis, primary hyperparathyroidism and chronic kidney disease. *Endocrine Reviews* 2018;**39**.

6. Bakides S, Papouli F, Zannou A, Soumbassis C, Kotis M, Dimaresi T*, et al.* Utility of frax algorithm and qus densitometry among women in an area of Southeastern Greece. *European Journal of Internal Medicine* 2011;**22:** S9-S10.

7. Balogun S, Winzenberg T, Wills K, Scott D, Callisaya M, Cicuttini F*, et al.* Prospective associations of osteosarcopenia and osteodynapenia with incident fracture and mortality over 10 years in community-dwelling older adults. *Arch Gerontol Geriatr* 2019;**82:** 67-73.

8. Brance ML, Larroudé MS, Brun LR. Study of risk factors for low bone mass in postmenopausal women from Argentina. *Journal of Clinical Rheumatology* 2018;**24:** S139-S140.

9. Buehring B, Lewis B, Hansen K, Cummings S, Lane N, Binkely N*, et al.* Association of musculoskeletal health score with fracture risk in the MrOS cohort. *Journal of Bone and Mineral Research* 2017;**31**.

10. Buehring B, Lewis BL, Hansen KE, Cummings SR, Lane NE, Ensrud KE*, et al.* Association of dysmobility syndrome with fracture risk inthe mros cohort. *Osteoporosis International* 2017;**28:** S290-S291.

11. Cantrell DC, Or K, Kumar EK, Koren SK, Mirovsky Y. Fragility fractures in patients admitted to assaf harofeh medical center: Clinical charact eristics and prefracture risk assessment. *Osteoporosis International* 2019;**30:** S351.

12. Chan DC, Tsai KS, Hwang JS, Yang RS. Osteoporosis awareness and 10 year probability of fracture from FRAX® among female breast cancer survivors. *Osteoporosis International* 2012;**23:** S149.

13. Chang SF, Yang RS. Optimal Analysis to Discriminate Males' Osteoporosis With Simple Physiological Indicators: A Cutoff Point Study. *Am J Mens Health* 2016;**10:** 487-494.

14. Christensen B, King BJ. The effect of mandibular fracture treatment on nutritional status. *Journal of Oral and Maxillofacial Surgery* 2016;**74:** e30.

15. Conversano F, Casciaro E, Greco A, Pisani P, Franchini R, Quarta E*, et al.* Evaluation of bone mineral density on femoral neck: Preliminary clinical validation of a new ultrasonic method. *Annals of the Rheumatic Diseases* 2013;**72**.

16. Conversano F, Pisani P, Greco A, Soloperto G, Muratore M, Casciaro S. An innovative ultrasound-based method for the estimation of osteoporotic fracture risk. *Annals of the Rheumatic Diseases* 2014;**73**.

17. Coyne M, Hoffmaster R, Sakely H. Identifying unintentional weight loss in a gerontology clinic. *Journal of the American Geriatrics Society* 2020;**68:** S210.

18. Davis WA, Hamilton EJ, Bruce DG, Davis TME. A hip fracture risk equation for type 2 diabetes: The Fremantle Diabetes Study. *Diabetologia* 2017;**60:** S112.

19. Deloumeau A, Moltó A, Roux C, Briot K. Determinants of short term fracture risk in patients with a recent history of low-trauma non-vertebral fracture. *Bone* 2017;**105:** 287-291.

20. Fernández-Ruiz M, Guerra-Vales JM, Trincado R, Medrano MJ, Benito-León J, Bermejo-Pareja F. Hip fracture in three elderly populations of central Spain: data from the NEDICES study. *Intern Emerg Med* 2014;**9:** 33-41.

21. Franic D, Verdenik I. Risk Factors for Osteoporosis in Postmenopausal Women - from The Point of View of Primary Care Gynecologist. *Zdr Varst* 2018;**57:** 33-38.

22. Fusaro M, Gallieni M, D'Arrigo G, Pitino A, Aghi A, Robinson BM*, et al.* PPI use is associated with higher prevalence of bone fractures and mortality in young hemodialysis patients. *Journal of the American Society of Nephrology* 2017;**28:** 915-916.

23. Gajic-Veljanoski O, Adachi JD, Kennedy C, Ioannidis G, Berger C, Wong AKO*, et al.* Vertebral fractures have similar impact as hip fractures on the progression of frailty. *Journal of Bone and Mineral Research* 2017;**31**.

24. Gold DT, Williams SA, Weiss RJ, Wang Y, Watkins C, Carroll J*, et al.* Quality of life in patients with osteoporosis: A US cross-sectional survey. *Value Health* 2018;**21:** S199-S200.

25. Gonzalez AP, Medina A. Prevalence of sarcopenia in patients with diagnosis of primary osteoporosis. *Osteoporosis International* 2018;**29:** S559-S560.

26. Gupta A, Kohn N, Sharma N, Vij B, Wolf-Klein G. Gender bias in diagnosis and management of osteoporosis. *Journal of the American Geriatrics Society* 2012;**60:** S122.

27. Handy CE, Desai CS, Dardari Z, Al-Mallah MH, Miedema M, Ouyang P*, et al.* The association of coronary artery calcium with age-related non-cardiovascular disease: The importance of “biologic aging” from the multi-ethnic study of atherosclerosis (MESA). *Circulation* 2014;**130**.

28. Homann S, Grijalva C, Tanner SB, Morrow J. Tumor necrosis factor alpha inhibitor (TNF-α inhibitor) exposure and risk of hip fracture in veterans with rheumatoid arthritis: A nested case control study. *Arthritis and Rheumatology* 2019;**71:** 2313-2314.

29. Honda C, Tomizawa R, Saeki S, Inui F, Sakai N. Investigation of environmental influence on bone health among Japanese female twins. *Behavior Genetics* 2019;**49:** 520.

30. Lalmohamed A, Welsing PMJ, Lems WF, Jacobs JWG, Kanis JA, Johansson H*, et al.* Calibration of FRAX ® 3.1 to the Dutch population with data on the epidemiology of hip fractures. *Osteoporosis International* 2012;**23:** 861-869.

31. Lee JS, Auyeung TW, Chau PP, Hui E, Chan F, Chi I*, et al.* Obesity can benefit survival-a 9-year prospective study in 1614 Chinese nursing home residents. *J Am Med Dir Assoc* 2014;**15:** 342-348.

32. Lee JSW, Auyeung TW, Chau PPH, Hui E, Chan F, Chi I*, et al.* Obesity can benefit survival-A 9-year prospective study in 1614 Chinese nursing home residents. *Journal of the American Medical Directors Association* 2014;**15:** 342-348.

33. Lim PN, Ooi LJ, Ong T, Neighbour C, Sahota O. Pelvic fractures in older people admitted to hospital: The clinical burden. *Age Ageing* 2018;**47:** iii9.

34. Lopez Gavilanez E, Johansson H, McCloskey E, Harvey NC, Segale Bajana A, Marriott Blum D*, et al.* Assessing the risk of osteoporotic fractures: the Ecuadorian FRAX model. *Arch Osteoporos* 2019;**14:** 93.

35. Mendoza ES, Lopez AA, Valdez VAU, Mercado-Asis LB. Osteoporosis and prevalent fractures among adult filipino men screened for bone mineral density in a tertiary hospital. *Endocrinology and Metabolism* 2016;**31:** 433-438.

36. Moberg L, Nilsson PM, Samsioe G, Sallsten G, Barregard L, Engström G*, et al.* Increased blood cadmium levels were not associated with increased fracture risk but with increased total mortality in women: the Malmö Diet and Cancer Study. *Osteoporosis International* 2017;**28:** 2401-2408.

37. Morrison CL, Purewal TS, Weston PJ. Diabetoporosis: Osteoporotic fragility fractures in patients with type 2 diabetes. *Diabetic Medicine* 2019;**36:** 140-141.

38. Muftic M, Selimovic EK, Miladinovic K. Osteoporosis--comparative study between quantitative ultrasound of calcaneus and DXA. *Med Arch* 2013;**67:** 289-291.

39. Naharci MI, Karadag B, Sahin S, Ozkaya H, Safer U, Curgunlu A*, et al.* Low vitamin D level in patients with alzheimer's disease (devit-alz): A crosssectional multicenter study. *Osteoporosis International* 2013;**24:** S127-S128.

40. Najar MS, Mir MM, Muzamil M. Prevalence of osteoporosis in patients with chronic kidney disease (stages 3-5) in comparison with age- and sex-matched controls: A study from Kashmir Valley Tertiary Care Center. *Saudi J Kidney Dis Transpl* 2017;**28:** 538-544.

41. Negm AM, Ioannidis G, Jantzi M, Bucek J, Giangregorio L, Pickard L*, et al.* Validation of a one year fracture prediction tool for absolute hip fracture risk in long term care residents. *BMC Geriatr* 2018;**18:** 320.

42. Obunwale A, Sloane R, Pieper C, Lee R, Lyles K, Cathleen CE*, et al.* Cholinesterase inhibitor use is associated with lower fracture risk in men with dementia. *Journal of Bone and Mineral Research* 2019;**34:** 24.

43. Patel V, Papamargaritis D, Francis J. Audit of the use of teriparatide in a tertiary centre. *Osteoporosis International* 2016;**27:** S679.

44. Povoroznyuk VV, Grygorieva NV, McCloskey EV, Johansson H, Kanis JA. Application of FRAX to determine the risk of osteoporotic fractures in the Ukrainian population. *International Journal of Osteoporosis and Metabolic Disorders* 2018;**11:** 7-13.

45. Rai M, Rai T, Dsa J, Rai S. Bone turnover markers; An emerging tool to detect primary osteoporosis. *Journal of Clinical and Diagnostic Research* 2018;**12:** BC04-BC07.

46. Reid I, Horne A, Mihov B, Stewart A, Garratt L, Bolland M*, et al.* Zoledronate every 18 months for 6 years in osteopenic postmenopausal women: effects on fractures and non-skeletal endpoints. *JBMR plus* 2018;**2:** S14‐.

47. Robinson D, Morgan R, Boutin R, Lenchik L. CT measured muscle density predicts 90-day mortality following hip fracture. *Skeletal Radiology* 2017;**45:** 420-421.

48. Roza NA, Quadros KR, Esteves AB, Franca RA, Borges CM, Carbonara CE*, et al.* Advanced glycation end-products (AGEs) accumulation and skeletal complications in CKD patients. *Journal of the American Society of Nephrology* 2018;**29:** 553.

49. Safai Haeri N, Baharlou S. Management of osteoporosis in older adults; a prognostic approach to a common scenario. *Journal of the American Geriatrics Society* 2018;**66:** S181-S182.

50. Sánchez-De Las Matas R, Fernández-García G. Osteoporosis and hip fracture: Risk factor's. *Osteoporosis International* 2016;**27:** S547-S548.

51. Schmid T, Brumme UM, Kemerle S, Zimmer K. Elderly osteoporosis suspects without diagnosis-interim data from a german geriatric practice. *Value Health* 2015;**18:** A654.

52. Shaul J, Favell D, Hill R, Huber B, Howe J, Bouxsein ML. Resorbable AGN1 biomaterial confers biomechanical integrity to osteoporotic cadaveric femurs. *Journal of Orthopaedic Research* 2017;**35**.

53. Sibilla F, Le Huec JC, Demezon H, Rigal J, Costes S. Safety and effectiveness of A new osteosynthesis device dedicated to prevent hip fracture. *HIP International* 2014;**24:** 520.

54. Skingle L, Poole K, Sano H, Turmezei T. A study of the microstructure of the femoral head in hip fracture. *Journal of Bone and Mineral Research* 2019;**34:** 290.

55. Sornay-Rendu E, Duboeuf F, Boutroy S, Chapurlat RD. Muscle mass is associated with incident fracture in postmenopausal women: The OFELY study. *Bone* 2017;**94:** 108-113.

56. Sta. Rosa Mendoza E, Mercado-Asis LB. Prevalent fractures occur at relatively younger age and higher bone mineral density among adult filipino men. *Endocrine Reviews* 2014;**35**.

57. Tanaka MF. Reframing refractoryanorexia nervosa as a chronic illness: A palliative approach. *Journal of General Internal Medicine* 2017;**32:** S582.

58. Tayal V, Nair JR, Abernethy R, Dawson J, Clewes A, O'Rourke T. Does addition of bone mineral density to FRAX analysis help reclassify fracture risk? *Annals of the Rheumatic Diseases* 2013;**72**.

59. Thein FS, Li Y, Nyunt MSZ, Gao Q, Wee SL, Ng TP. Physical frailty and cognitive impairment is associated with diabetes and adversely impact functional status and mortality. *Postgraduate Medicine* 2018;**130:** 561-567.

60. Thornqvist C, Gislason GH, Køber L, Jensen PF, Torp-Pedersen C, Andersson C. Body mass index and risk of perioperative cardiovascular adverse events and mortality in 34,744 Danish patients undergoing hip or knee replacement. *Acta Orthopaedica* 2014;**85:** 456-462.

61. Tseng WJ, Hung LW, Shieh JS, Abbod MF, Lin J. Hip fracture risk assessment: artificial neural network outperforms conditional logistic regression in an age- and sex-matched case control study. *BMC Musculoskelet Disord* 2013;**14:** 207.

62. Vaswani R, Manoli A, Goch A, Egol KA. Surgical Fracture Repair in Chronic Renal Failure Patients on Hemodialysis An Analysis of Complications and Hospital Quality Measures. *Bull Hosp Jt Dis (2013)* 2016;**74:** 160-164.

63. Vitiello R, Perisano C, Covino M, Perna A, Bianchi A, Oliva MS*, et al.* Euthyroid sick syndrome in hip fractures: Valuation of vitamin D and parathyroid hormone axis. *Injury* 2020;**51 Suppl 3:** S13-s16.

64. White M, Barre L. Aromatase Inhibitor-Associated Bone Loss: Screening and Prevention. *Arthritis and Rheumatology* 2020;**72:** 224-226.

65. Wiklund R, Conradsson M, Gustafson Y, Littbrand H, Olofsson B, Rosendahl E*, et al.* Risk factors for hip fractures in very old people: A population-based study. *Physiotherapy (United Kingdom)* 2015;**101:** eS891-eS892.

66. Wong KTE, Ho KWK, Dai D, Hung LK, Chan KS, Chan KLG. Preventive measure of second hip fracture: Identification of high risk group. *Physiotherapy (United Kingdom)* 2015;**101:** eS1655.

67. Woods GN, Huang MH, Lee JH, Cawthon PM, Fink HA, Schousboe JT*, et al.* Factors Associated With Kyphosis and Kyphosis Progression in Older Men: The MrOS Study. *J Bone Miner Res* 2020;**35:** 2193-2198.

68. Zullo AR, Lee Y, Daiello LA, Zhang T, McConeghy KW, Kiel DP*, et al.* Predictors of hip fracture among frail older adults treated with bisphosphonates. *Journal of the American Geriatrics Society* 2019;**67:** S144.

69. Abuín-Fernández J, Pérez-del Río V, García-Olivares M, Porras-Perez N, Bravo-Bardají M, García de Quevedo-Puerta D*, et al.* Nutritional assessment in fragility hip fracture. *Clinical Nutrition ESPEN* 2020;**40:** 474.

70. Anghel D, Otlocan L, Negru MM, Busuioc E, Bursuc R, Smedescu V*, et al.* Fracture risk assessment (FRAX) in patients with rheumatoid arthritis in therapy with anti-TNFα. *Osteoporosis International* 2019;**30:** S400.

71. Anthony CA, Bedard NA, Duchman KR, Pugely AJ, Gao Y, Callaghan JJ. Hip fractures treated with total hip arthroplasty: Incidence and risk factors for surgical delay. *Journal of Orthopaedic Research* 2017;**35**.

72. Arkley J, Taher S, Dixon J, Dietz-Collin G, Wales S, Wilson F*, et al.* Too Cool? Hip Fracture Care and Maintaining Body Temperature. *Geriatr Orthop Surg Rehabil* 2020;**11:** 2151459320949478.

73. Arshi A, Lai WC, Iglesias BC, McPherson EJ, Zeegen EN, Stavrakis AI*, et al.* Blood transfusion rates and predictors following geriatric hip fracture surgery. *Hip Int* 2021;**31:** 272-279.

74. Atreya AR, Arora S, Brennan MJ. Hip fractures in elders nearing end of life: Is there a role for palliative surgery? *Journal of the American Geriatrics Society* 2012;**60:** S136-S137.

75. Bell JJ, Bauer JD, Capra S, Pulle CR. Concurrent and predictive evaluation of malnutrition diagnostic measures in acute hip fracture inpatients. *Clinical Nutrition* 2013;**32:** S74.

76. Bell JJ, Bauer JD, Capra S, Pulle RC. Concurrent and predictive evaluation of malnutrition diagnostic measures in hip fracture inpatients: a diagnostic accuracy study. *Eur J Clin Nutr* 2014;**68:** 358-362.

77. Binkley N, Hare K, Krueger D, Borchardt G, Anderson P. Periprosthetic fractures: The next osteoporosis crisis. *Journal of Bone and Mineral Research* 2019;**34:** 248.

78. Boutin R, Bamrungchart S, Bateni C, Beavers K, Beavers D, Meehan J*, et al.* CT of hip fracture patients: Canmuscle size& attenuation predict clinical outcomes? *Skeletal Radiology* 2016;**45:** 1167-1168.

79. Catania P, Passaretti D, Montemurro G, Ripanti S, Carbone S, Candela V*, et al.* Intramedullary nailing for pertrochanteric fractures of proximal femur: a consecutive series of 323 patients treated with two devices. *J Orthop Surg Res* 2019;**14:** 449.

80. Chandran M, Tay D, Huang XF, Hao Y. The burden of inpatient care for diabetic and non-diabetic patients with osteoporotic hip fractures—does it differ? An analysis of patients recruited into a fracture liaison service in Southeast Asia. *Archives of Osteoporosis* 2018;**13**.

81. Chang CD, Wu JS, Mhuircheartaigh JN, Hochman MG, Rodriguez EK, Appleton PT*, et al.* Effect of sarcopenia on clinical and surgical outcome in elderly patients with proximal femur fractures. *Skeletal Radiol* 2018;**47:** 771-777.

82. Chen S, Jain M, Jhangiani S, Akdemir ZC, Campeau PM, Klein RF*, et al.* Genetic Burden Contributing to Extremely Low or High Bone Mineral Density in a Senior Male Population From the Osteoporotic Fractures in Men Study (MrOS). *JBMR Plus* 2020;**4:** e10335.

83. Chen Sr YC. Pre-operative magnetic resonance imaging can help in predicting two-year readmission in acute severe osteoporotic vertebral fracture after vertebroplasty vertebroplasty. *Arthritis and Rheumatology* 2016;**68:** 445-447.

84. Chen YP, Wong PK, Tsai MJ, Chang WC, Hsieh TS, Leu TH*, et al.* The high prevalence of sarcopenia and its associated outcomes following hip surgery in Taiwanese geriatric patients with a hip fracture. *J Formos Med Assoc* 2020;**119:** 1807-1816.

85. ChiCtr. A randomized controlled trial for the efficacy of early analgesia by continuous fascia block under ultrasound guidance for elderly patients with hip fracture. 2018.

86. Crawford ZT, Southam B, Matar R, Avilucea FR, Bowers K, Altaye M*, et al.* A Nomogram for Predicting 30-day Mortality in Elderly Patients Undergoing Hemiarthroplasty for Femoral Neck Fractures. *Geriatr Orthop Surg Rehabil* 2020;**11:** 2151459320960087.

87. Del Rosario Evangelista Cabrera L, Lueje Alonso E, Garay Airaghi V, Fernández Arana L, Mora Fernández J. Handgrip strength as a predictor of functional recovery in elderly patients with hip fracture. *European Geriatric Medicine* 2017;**8:** S103-S104.

88. Evangelista L, Cuesta F, Matía P, Fernández L, Garay V, Mora J. Sarcopenia and nutritional status in elderly patients with fragility hip fracture. *European Geriatric Medicine* 2016;**7:** S118.

89. Fakler JK, Grafe A, Dinger J, Josten C, Aust G. Perioperative risk factors in patients with a femoral neck fracture - influence of 25-hydroxyvitamin D and C-reactive protein on postoperative medical complications and 1-year mortality. *BMC Musculoskelet Disord* 2016;**17:** 51.

90. Fakler JKM, Grafe A, Dinger J, Josten C, Aust G. Perioperative risk factors in patients with a femoral neck fracture - Influence of 25-hydroxyvitamin D and C-reactive protein on postoperative medical complications and 1-year mortality Epidemiology of musculoskeletal disorders. *BMC Musculoskelet Disord* 2016;**17**.

91. Gibbs VN, McCulloch RA, Dhiman P, McGill A, Taylor AH, Palmer AJR*, et al.* Modifiable risk factors for mortality in revision total hip arthroplasty for periprosthetic fracture. *Bone Joint J* 2020;**102-b:** 580-585.

92. Hansson S, Nemes S, Kärrholm J, Rogmark C. Reduced risk of reoperation after treatment of femoral neck fractures with total hip arthroplasty. *Acta Orthop* 2017;**88:** 500-504.

93. Hao L, Carson JL, Schlussel Y, Noveck H, Shapses SA. Vitamin D deficiency is associated with reduced mobility after hip fracture surgery: a prospective study. *Am J Clin Nutr* 2020;**112:** 613-618.

94. Hassan BK, Sahlström A, Dessau RB. Risk factors for renal dysfunction after total hip joint replacement; a retrospective cohort study. *J Orthop Surg Res* 2015;**10:** 158.

95. Hoyos R, González-Montalvo JI, Alarcón T, Pallardo B, Gotor P, Otero A*, et al.* Comparison of two European Working Group on Sarcopenia in Older People (EWGSOP) handgrip strength cutoffs on a series of acute hip fracture patients. *European Geriatric Medicine* 2013;**4:** S57.

96. Irgit K, Richard RD, Cornelius A, Bowen TR, Andreychik CM, Horwitz DS. Osteosynthesis compared to hemiarhro plasty for osteoporotic, undisplaced and stable femoral neck fractures. *Osteoporosis International* 2013;**24:** S427-S428.

97. Jaller-Raad JJ, Jaller-Char JJ, Lechuga-Ortiz JA, Navarro-Lechuga E, Johansson H, Kanis JA. Incidence of hip fracture in Barranquilla, Colombia, and the development of a Colombian FRAX model. *Calcif Tissue Int* 2013;**93:** 15-22.

98. James H, Holte P, Kearns AE. Vitamin D deficiency in hip fracture patients: Prevalence and a quality improvement initiative to improve screening and treatment. *Endocrine Reviews* 2014;**35**.

99. Johansson H, Adib G, McCloskey EV, Harvey NC, Lorentzon M, Liu E*, et al.* Ten-year fracture probability in syria using a surrogate model. *Osteoporosis International* 2019;**30:** S489.

100. Johansson H, Matijevic R, Harhaji V, McCloskey EV, Harvey NC, Lorentzon M*, et al.* Ten-year fracture probability in serbia according to age and other FRAX models in the region. *Osteoporosis International* 2019;**30:** S489-S490.

101. Johnson KC, Bray GA, Cheskin LJ, Clark JM, Egan CM, Foreyt JP*, et al.* The Effect of Intentional Weight Loss on Fracture Risk in Persons With Diabetes: Results From the Look AHEAD Randomized Clinical Trial. *Journal of Bone and Mineral Research* 2017;**32:** 2278-2287.

102. Juliebø V, Krogseth M, Neerland BE, Watne LO, Wyller TB. Delirium - a common condition associated with negative outcome in the elderly. *Norsk Epidemiologi* 2012;**22:** 253-260.

103. Kang JH, Chung HK, Baik HW. Changes of antioxidant capacity after hip fracture in Korean elderly patients. *Clinical Nutrition* 2013;**32:** S239.

104. Kim ES, Kim CH. Hip fractures in patients with Parkinson's disease. *Journal of Parkinson's Disease* 2019;**9:** 248.

105. Lambert NA, Hans DH, Heinzer R, Haba-Rubio J, Marques-Vidal P, Lamy O. Sleep disorders are associated with trabecular bone score and osteoporotic fracture, not with bone mineral density. *Osteoporosis international* 2017;**28:** S372‐.

106. Lee GH, Lim JW, Park YG, Ha YC. Vitamin D Deficiency Is Highly Concomitant but Not Strong Risk Factor for Mortality in Patients Aged 50 Year and Older with Hip Fracture. *J Bone Metab* 2015;**22:** 205-209.

107. Leslie W, Morin S, Lix L, McCloskey E, Johansson H, Harvey N*, et al.* Fracture risk assessment in women with breast cancer initiating aromatase inhibitor therapy: A registry-based cohort study. *Journal of Bone and Mineral Research* 2018;**33:** 47-48.

108. Lim JW, Ng GS, Jenkins RC, Ridley D, Jariwala AC, Sripada S. Total hip replacement for neck of femur fracture: Comparing outcomes with matched elective cohort. *Injury* 2016;**47:** 2144-2148.

109. Lu WL, Kumar DS, Lin WL, Shen HM, Cheng Q, Zheng SB. Pre-and post-operative risk and post-operative evaluation for hip fracture in the elderly. *Journal of the American Geriatrics Society* 2014;**62:** S394.

110. Lundin H, Sääf M, Strender LE, Nyren S, Johansson SE, Salminen H. High Serum Insulin-Like Growth Factor-Binding Protein 1 (IGFBP-1) is Associated with High Fracture Risk Independent of Insulin-Like Growth Factor 1 (IGF-I). *Calcif Tissue Int* 2016;**99:** 333-339.

111. Lüthje P, Nurmi-Lüthje I, Tavast N, Villikka A, Kataja M. Evaluation of minimal fracture liaison service resource: costs and survival in secondary fracture prevention—a prospective one-year study in South-Finland. *Aging Clinical and Experimental Research* 2021.

112. Mahon J, Hannigan O, Steen G, Fallon N, Maher N, Dillon A*, et al.* Characteristics and consequences of hip fracture in an elderly irish population. *Journal of Bone and Mineral Research* 2017;**32:** S154.

113. Martin I, Aparicio M, López-Vives L, Jordana M, Coscujuela A, Montero A*, et al.* Study on the relationship between the preoperative stay and morbimortality in patients with hip fracture. *Arthritis and Rheumatism* 2013;**65:** S524.

114. Martin-Esteve I, Aparicio M, Lopez-Vives L, Coscujuela A, Montero A, Gómez-Vaquero C. Prognostic factors of morbimortality in patients with hip fracture. *Annals of the Rheumatic Disease* 2013;**71**.

115. Martin-Esteve I, Aparicio M, Lopez-Vives L, Jordana M, Coscujuela A, Montero A*, et al.* Study on the relationship between the preoperative stay and morbimortality in patients with hip fracture. *Annals of the Rheumatic Diseases* 2013;**72**.

116. McCloskey E, Chiu S, Lenaghan E, Shepstone L, Birrell F, Harvey N. Impact of falls on fractures and mortality-an opportunity for intervention and enhancement of fracture prediction? *Osteoporosis International* 2016;**27:** S617.

117. Merello-De-Miguel A, Miret-Corchado C, Sanchez-Castellano C, Vaquero-Pinto MN, Ramirez-Archundia AC, Cruz-Jentoft AJ. Sarcopenia does not predict one-year mortality after a hip fracture. *Osteoporosis International* 2017;**28:** S68-S69.

118. Montanari L, Martini E, Conti F, Sandri S, Laus M, Lunardelli ML*, et al.* Direct oral anticoagulants can delay surgery in comparison with vitamin K antagonists and antiplatelet agents in hip fracture: The ortho-ger-DOAC study. *Blood Transfusion* 2018;**16:** s488.

119. Nct. Postoperative Intervention Program Effectiveness in Hip Fracture Patients: a Randomized Clinical Trial. 2017.

120. Ogawa T, Sato K, Nakayama Y, Nakazato A, Zaha M, Isa A*, et al.* Factors associated with actual skeletal muscle mass increase during hip fracture rehabilitation of persons aged 80 and older. *Arch Gerontol Geriatr* 2022;**98:** 104566.

121. Ogawa T, Yoshii T, Morishita S, Moriwaki M, Okawa A, Nazarian A*, et al.* Seasonal impact on surgical site infections in hip fracture surgery: Analysis of 330,803 cases using a nationwide inpatient database. *Injury* 2021;**52:** 898-904.

122. Or O, Lane JM, Halawa O, Marty E, Sayied R, Liu Y*, et al.* Does promis29 correlate with frailty in hip fracture patients? *Journal of Bone and Mineral Research* 2017;**32:** S287.

123. Paruk F, Cassim B, Matthews G. Predictors of mortality in hip fracture subjects aged 60 years and over with minimal trauma hip fractures in the ethekwini municipality, Kwazulu-Natal, SA. *Osteoporosis International* 2015;**26:** S106-S107.

124. Paul TV, Selvan SA, Asha HS, Thomas N, Venkatesh K, Oommen AT*, et al.* Hypovitaminosis D and other risk factors of femoral neck fracture in South Indian postmenopausal women: A pilot study. *Journal of Clinical and Diagnostic Research* 2015;**9:** OC19-OC22.

125. Petrusic T, Mandic N. The prevalence of osteoporotic fractures in the elderly population. *Osteoporosis International* 2014;**25:** S271.

126. Reina N, Delaunay C, Chiron P, Ramdane N, Hamadouche M. Infection as a cause of primary total hip arthroplasty revision and its predictive factors. *Orthop Traumatol Surg Res* 2013;**99:** 555-561.

127. Rosas J, Cano C, Lorente M, Pons A, Salas E, Llinares T*, et al.* Local fracture liaison service (fls): characteristics and first 6 months of evolution in patients after hip fracture: preliminary results. *Annals of the Rheumatic Diseases* 2017;**76:** 1351.

128. Ross LA, Keenan OJF, Magill M, Brennan CM, Clement ND, Moran M*, et al.* Management of low periprosthetic distal femoral fractures. *Bone Joint J* 2021;**103-b:** 635-643.

129. Ross RD, Shah RC, Leurgans SE, Buchman AS, Bennett DA. Association of Heel Bone Mineral Density With Incident Disability and Mortality in Community-Dwelling Older Adults. *JBMR Plus* 2020;**4:** e10390.

130. Ryan G, Nowak L, Melo L, Ward S, Atrey A, Schemitsch EH*, et al.* Anemia at Presentation Predicts Acute Mortality and Need for Readmission Following Geriatric Hip Fracture. *JB JS Open Access* 2020;**5**.

131. S S, B Y, W C, J B, M T, A S. Short versus long cephalomedullary nailing of pertrochanteric hip fractures: a randomized prospective study. *Orthopaedic trauma association annual meeting* 2018.

132. Sakao Y, Ojima T, Yasuda H, Hashimoto S, Hasegawa T, Iseki K*, et al.* Serum Creatinine Modifies Associations between Body Mass Index and Mortality and Morbidity in Prevalent Hemodialysis Patients. *PLoS One* 2016;**11:** e0150003.

133. Sánchez-Castellano C, Merello-De-Miguel A, Vaquero-Pinto MN, Ramírez-Archundia AC, Cruz-Jentoft AJ. Sarcopenia does not predict one-year-mortality after a hip fracture. *European Geriatric Medicine* 2016;**7:** S127-S128.

134. Sarli MJ, Firpo CG, Parisi C, Guerra JL, Garcia Basavilbaso NX, Baña F*, et al.* Bone mineral density, fracture risk and associated factors in argentinian type 2 diabetes mellitus patients aged over 55 years. *Osteoporosis International* 2016;**27:** S84.

135. Sathiyakumar V, Greenberg SE, Molina CS, Thakore RV, Obremskey WT, Sethi MK. Hip fractures are risky business: an analysis of the NSQIP data. *Injury* 2015;**46:** 703-708.

136. Schaefer MS, Hammer M, Platzbecker K, Santer P, Grabitz SD, Murugappan KR*, et al.* What Factors Predict Adverse Discharge Disposition in Patients Older Than 60 Years Undergoing Lower-extremity Surgery? The Adverse Discharge in Older Patients after Lower-extremity Surgery (ADELES) Risk Score. *Clin Orthop Relat Res* 2021;**479:** 546-547.

137. Schaller F, Sidelnikov E, Theiler R, Egli A, Staehelin HB, Dick W*, et al.* Mild to moderate cognitive impairment is a major risk factor for mortality and nursing home admission in the first year after hip fracture. *Bone* 2012;**51:** 347‐352.

138. Sedighinejad A, Naderi Nabi B, Ettehad H, Mirbolook A, Atrkarroushan Z, Ghazanfar Tehran S*, et al.* Does Adding Lidocaine to Intrathecal Bupivacaine Affect Hemodynamic Parameters during Hip Fracture Surgery? *Arch Bone Jt Surg* 2018;**6:** 390-396.

139. Sezgin EA, Tor AT, Markevičiūtė V, Širka A, Tarasevičius Š, Raina DB*, et al.* A combined fracture and mortality risk index useful for treatment stratification in hip fragility fractures. *Jt Dis Relat Surg* 2021;**32:** 583-589.

140. Shah A, Prieto-Alhambra D, Hawley S, Delmestri A, Lippett J, Cooper C*, et al.* Geographic variation in secondary fracture prevention after a hip fracture during 1999–2013: a UK study. *Osteoporosis International* 2017;**28:** 169-178.

141. Shiomoto K, Babazono A, Harano Y, Fujita T, Jiang P, Kim SA*, et al.* Effect of body mass index on vertebral and hip fractures in older people and differences according to sex: a retrospective Japanese cohort study. *BMJ Open* 2021;**11:** e049157.

142. Sieber F, Neufeld KJ, Gottschalk A, Bigelow GE, Oh ES, Rosenberg PB*, et al.* Depth of sedation as an interventional target to reduce postoperative delirium: mortality and functional outcomes of the Strategy to Reduce the Incidence of Postoperative Delirium in Elderly Patients randomised clinical trial. *Br J Anaesth* 2019;**122:** 480-489.

143. Sieber F, Neufeld KJ, Gottschalk A, Bigelow GE, Oh ES, Rosenberg PB*, et al.* Depth of sedation as an interventional target to reduce postoperative delirium: mortality and functional outcomes of the Strategy to Reduce the Incidence of Postoperative Delirium in Elderly Patients randomised clinical trial. *British Journal of Anaesthesia* 2019;**122:** 480-489.

144. Silva L, Santos M, Moura R, Oliveira A, Veríssimo R. Nutritional assessment in a Portuguese orthogeriatric unit. *European Geriatric Medicine* 2020;**11:** S258.

145. Sim SD, Sim YE, Tay K, Howe TS, Png MA, Chang CCP*, et al.* Preoperative hypoalbuminemia: Poor functional outcomes and quality of life after hip fracture surgery. *Bone* 2021;**143:** 115567.

146. Slatnick L, Maalouf N, Nguyen BN, Dolezal C. Determinants of calcium supplementation at discharge from inpatient rehabilitation after hip fracture. *PM and R* 2014;**6:** S220.

147. Slatnick L, Maalouf N, Nguyen BN, Dolezal C. Functional independence measure scores fail to identify patients who die following hip fracture. *PM and R* 2014;**6:** S220.

148. Smolle MA, Hörlesberger N, Maurer-Ertl W, Puchwein P, Seibert FJ, Leithner A. Periprosthetic fractures of hip and knee–A morbidity and mortality analysis. *Injury* 2021.

149. Steihaug OM, Gjesdal CG, Bogen B, Kristoffersen MH, Lien G, Ranhoff AH. Sarcopenia in patients with hip fracture: A multicenter cross-sectional study. *PLoS One* 2017;**12:** e0184780.

150. Sun CS, Zheng X, Guo KJ, Li DY, Li CY, Wang Y*, et al.* Relationship between low basal metabolic rate and mortality in older adults with hip fractures. *Chinese Journal of Tissue Engineering Research* 2018;**22:** 2467-2471.

151. Svenøy S, Westberg M, Figved W, Valland H, Brun OC, Wangen H*, et al.* Posterior versus lateral approach for hemiarthroplasty after femoral neck fracture: Early complications in a prospective cohort of 583 patients. *Injury* 2017;**48:** 1565-1569.

152. Syed Ahmad Kabeer SJ, Mohd Farid M, Nur Atiqah MJ, Zulaikha Z, Mohammad Rostam S, Ikhwan S*, et al.* Correlation of FRAX risk score and hip fragility fractures in elderly patients. *Malaysian Orthopaedic Journal* 2018;**12**.

153. Szpalski M, Jean-Charles L, Sibilla F, Costes S, Rigal J, Vienney C. Evaluation of a new prevention dedicated osteosynthesis device indicated to prevent hip fracture. *Osteoporosis International* 2014;**25:** 584.

154. Szpalski M, Le Huec JC, Sibilla F, Costes S, Winzenreith R, Vienney C. Impact evaluation ofa hip fracture prevention device. *Osteoporosis International* 2014;**25:** S161-S162.

155. Tctr. A comparison of efficacy between low dose dexmedetomidine and propofol for prophylaxis postoperative delirium in elderly patients undergoing fracture hip surgery. 2018.

156. Thörling J, Ljungqvist O, Sköldenberg O, Hammarqvist F. No association between preoperative impaired glucose control and postoperative adverse events following hip fracture surgery - A single-centre observational cohort study. *Clin Nutr* 2021;**40:** 1348-1354.

157. Udombhornprabha A, Boonhong J, Tejapongvorachai T, Komoltri C, Sermsri S. Quality of life for thai hip fracture patients: Assessments with medical outcomes study, A 36-item short form survey (MOS SF-36) and one-year health care resource utilization in a public hospital. *Value Health* 2011;**14:** A314.

158. Van Haecke A, Viste A, Desmarchelier R, Roy P, Mercier M, Fessy MH. Incidence and risk factors for bilateral proximal femoral fractures. *Orthop Traumatol Surg Res* 2021**:** 102887.

159. van Laarhoven SN, Vles GF, van Haaren EH, Schotanus MGM, van Hemert WLW. Tapered, fluted, modular, titanium stems in Vancouver B periprosthetic femoral fractures: an analysis of 87 consecutive revisions. *Hip Int* 2020**:** 1120700020904933.

160. Wactawski-Wende J, Larson JC, Cauley J, Chen Z, Jackson R, LaCroix A*, et al.* Physical activity and incident fracture in postmenopausal women: The women's health initiative observational study. *Journal of Bone and Mineral Research* 2012;**27**.

161. Watanabe H, Minagawa Y, Suzuki I, Kitamura K, Watanabe Y, Kabasawa K*, et al.* Secular changes in bone mineral density of adult Japanese women from 1995 to 2013. *Fukushima J Med Sci* 2021.

162. Wise BL, Parimi N, Zhang Y, Cawthon PM, Barrett-Connor E, Ensrud K*, et al.* Elderly men with hip osteoarthritis are frail and frailty remains after total hip replacement in the mros cohort. *Osteoarthritis and Cartilage* 2011;**19:** S154.

163. Yang RS, Chao CT, Huang WJ, Chan DC. A multicentre cohort study of risk factors for mortality, falls, and recurrent fractures among patients under fracture liaison service. *Osteoporosis International* 2019;**30:** S82.

164. Yang RS, Yang TH, Huang WJ, Chan DC. Impact of medication use pattern on 2-year outcomes of fracture liaison service patients. *Osteoporosis International* 2019;**30:** S374.

165. Yong E. Hip fractures and mediators of low bone mineral density in multi-ethnic Singapore women. *Osteoporosis International* 2019;**30:** S449.

166. Zerbini CAF, Szejnfeld VL, Abergaria BH, McCloskey EV, Johansson H, Kanis JA. Incidence of hip fracture in Brazil and the development of a FRAX model. *Archives of Osteoporosis* 2015;**10**.

167. Ziebart C, Adachi JD, Ashe MC, Bleakney RR, Cheung AM, Gibbs JC*, et al.* Exploring the association between number, severity, location of fracture, and occiput-to-wall distance. *Arch Osteoporos* 2019;**14:** 27.

168. Nissen FI, Andreasen C, Borgen TT, Bjørnerem Å, Hansen AK. Cortical bone structure of the proximal femur and incident fractures. *Bone* 2021**:** 116284.

169. Prieto-Alhambra D, Tebé C, Pallisó F, Judge A, Arden NK, Cooper C*, et al.* Predictors of post-operative mortality following total hip arthroplasty surgery: A population-based cohort study. *Osteoporosis International* 2015;**26:** S44-S45.

170. Scully W, Piuzzi NS, Sodhi N, Sultan AA, George J, Khlopas A*, et al.* The effect of body mass index on 30-day complications after total hip arthroplasty. *HIP International* 2020;**30:** 125-134.

171. Vosoughi AR, Emami MJ, Pourabbas B, Mahdaviazad H. Factors increasing mortality of the elderly following hip fracture surgery: role of body mass index, age, and smoking. *Musculoskelet Surg* 2017;**101:** 25-29.

172. Antwi F, Fazylova N, Garcon MC, Lopez L, Rubiano R, Slyer JT. The effectiveness of web-based programs on the reduction of childhood obesity in school-aged children: A systematic review. *JBI Libr Syst Rev* 2012;**10:** 1-14.

173. Barake M, El Eid R, Ajjour S, Chakhtoura M, Meho L, Mahmoud T*, et al.* Osteoporotic hip and vertebral fractures in the Arab region: a systematic review. *Osteoporos Int* 2021.

174. Barake M, El Eid R, Chakhtoura M, Meho L, Mahmoud T, Atieh J*, et al.* Osteoporotic Fractures in the Arab region A Systematic Review. *Journal of Bone and Mineral Research* 2020;**35:** 222.

175. Kanis J. Getting to grips with Frax®: How it really works. *Osteoporosis International* 2011;**22:** S505-S506.

176. Kelly M, Zhang J, Ackert-Bicknell C, Zusick M. Effect of ovariectomy and high fat diet on the murine femur. *Journal of Orthopaedic Research* 2017;**35**.

177. McGowan B, Silke C, Whelan B. The development of the FRAX® model for Ireland. *Osteoporosis International* 2013;**24:** S304-S305.

178. Micic D. What are the risk of weight loss in the elderly? *Obesity Reviews* 2011;**12:** 3.

179. Oduoza U, Mamarelis G, Chekuri R, Estfan R, Greer A. SARS-cov-2 increases the 30-day mortality in hip fracture patients. A uk district hospital's experience. *Br J Surg* 2021;**108:** ii23.

180. Olatokun O, Nash T, Abdul-Jabar HB. A rare case of neck of femur fracture in a female adolescent associated with minor trauma and impaired bone metabolism. *JBMR Plus* 2018;**2:** S44-S45.

181. Tomás CC, Oliveira E, Sousa D, Uba-Chupel M, Furtado G, Rocha C*, et al.* Proceedings of the 3rd IPLeiria's International Health Congress : Leiria, Portugal. 6-7 May 2016. *BMC Health Serv Res* 2016;**16 Suppl 3:** 200.

182. Artaza I, Urkiza M, Martinez V, Aurre I, Mendieta M, Elgezua E. Determining factors of mortality in a geriatric rehabilitation unit after a hip fracture surgery. *European Geriatric Medicine* 2011;**2:** S114.

183. Boutin E, Natella PA, Schott AM, Bastuji-Garin S, David JP, Paillaud E*, et al.* Interrelations between body mass index, frailty, and clinical adverse events in older community-dwelling women: The EPIDOS cohort study. *Clin Nutr* 2018;**37:** 1638-1644.

184. Brites L, Marques M, Daniel A, Santiago M, Marques A, Pereira Da Silva J. Predictors of mortality and re-fracture at 1 and 3years after hip fracture. *Annals of the Rheumatic Diseases* 2017;**76:** 700-701.

185. Chen CH, Wang HY, Huang HT, Lu YM, Chen JC, Lin SY*, et al.* Care of elderly patients with hip fragility fracture by specialized hip surgeons and treatment of underlying disease after discharge reduces one-year mortality. *Osteoporosis International* 2019;**30:** S94-S95.

186. Daugaard C, Kristensen NR, Pedersen AB, Johnsen SP. Preadmission antithrombotic therapy and risk of blood transfusion and 30-days mortality following hip fracture surgery: A Danish nationwide cohort study. *Pharmacoepidemiology and Drug Safety* 2018;**27:** 289.

187. Deren ME, Babu J, Cohen EM, Machan J, Born CT, Hayda R. Increased Mortality in Elderly Patients with Sarcopenia and Acetabular Fractures. *J Bone Joint Surg Am* 2017;**99:** 200-206.

188. Geirsdottir OG, Ramel A, Chang M, Jonsson PV, Thorsdottir I. Healthy range of BMI in old adults. *European Geriatric Medicine* 2020;**11:** S253.

189. Gill LE, Bartels SJ, Batsis JA. Weight Management in Older Adults. *Curr Obes Rep* 2015;**4:** 379-388.

190. Guerra M, Ganhão S, Aguiar F, Terroso G, Vieira R, Goncalves D*, et al.* Predictors of mortality in patients with hip fragility fracture. *Arthritis and Rheumatology* 2018;**70:** 2567-2568.

191. Hori K, Siu AM, Nguyen ET, Andrews SN, Choi SY, Ahn HJ*, et al.* Osteoporotic hip fracture mortality and associated factors in Hawai'i. *Arch Osteoporos* 2020;**15:** 183.

192. Kawińska-Hamala A, Kawiński A, Stanek K, Stuss M, Sewerynek E. Correlations between 10-year risk of death from cardiovascular diseases. *Endokrynol Pol* 2017;**68:** 390-397.

193. Kawińska-Hamala A, Kawiński A, Stanek K, Stuss M, Sewerynek E. Correlations between 10-year risk of death from cardiovascular diseases and 10-year osteoporotic fracture risk in postmenopausal women. *Endokrynologia Polska* 2017;**68:** 390-397.

194. Kougioumtzis IE, Tottas S, Tilkeridis K, Ververidis A, Drosos GI. Elderly patients with hip fractures, treatment for osteoporosis, evidence for sarcopenia and malnutrition. A preliminary report. *Journal of Musculoskeletal Neuronal Interactions* 2021;**21:** 185-186.

195. Lafage-Proust MH. Management of osteoporosis in renal failure. *Osteoporosis International* 2015;**26:** S29-S30.

196. Lee JSW, Auyeung T, Chau PPH, Hui E, Chan F, Chow KKM*, et al.* Is body mass index or weight loss more important in survival? A 9-year prospective study in 1,816 nursing home residents. *Journal of Cachexia, Sarcopenia and Muscle* 2011;**2:** 242.

197. Levi A, Coste M, Shah NV, Warshowsky E, Harry F, Lavery R*, et al.* Cracking the Hip: Does Protocol Matter? *J Am Coll Surg* 2020;**231:** S311.

198. Liao CY, Tan TL, Lu YD, Wu CT, Lee MS, Kuo FC. Does preoperative dipyridamole-thallium scanning reduce 90-day cardiac complications and 1-year mortality in patients with femoral neck fractures undergoing hemiarthroplasty? *J Orthop Surg Res* 2020;**15:** 385.

199. Lindberg-Larsen M, Petersen PB, Jørgensen CC, Overgaard S, Kehlet H. Postoperative 30-day complications after cemented/hybrid versus cementless total hip arthroplasty in osteoarthritis patients > 70 years. *Acta Orthop* 2020;**91:** 286-292.

200. Mlot-Michalska M, Grzegorzewska AE. Ten-year fracture risk calculated with Bone Mineral Density (BMD) or Body Mass Index (BMI) only as mortality predictor in dialyzed patients. *Peritoneal Dialysis International* 2012;**32:** S7.

201. Murena L, Ratti C, Maritan G, Rasio N, Pistorio S, Cusitore M*, et al.* Epidemiology and risk factors for contralateral proximal femur fracture: a single center retrospective cohort study on 1022 patients. *Acta Biomed* 2020;**91:** 115-121.

202. Yamauchi Y, Yasunaga H, Sakamoto Y, Hasegawa W, Takeshima H, Urushiyama H*, et al.* Mortality associated with bone fractures in COPD patients. *Int J Chron Obstruct Pulmon Dis* 2016;**11:** 2335-2340.

203. Yang B, Graver AJ, Merwin S, Collins L, Nellans K, Goldman A. Effects of preoperative nutritional status on 30-day mortality in patients undergoing surgery for hip fractures. *J Am Coll Surg* 2014;**219:** S70-S71.

204. Zaslavsky O, Zelber Sagi S. Frailty and body composition: Results from the women's health initiative study. *Journal of the American Geriatrics Society* 2017;**65:** S136.

205. Bell JJ, Pulle RC, Lee HB, Ferrier R, Crouch A, Whitehouse SL. Diagnosis of overweight or obese malnutrition spells DOOM for hip fracture patients: A prospective audit. *Clin Nutr* 2021;**40:** 1905-1910.

206. DeVries F, Leufkens HGM, Bijlsma J, Elders P, Harvey NC, Cooper C*, et al.* Mortality in british hip fracture patients, 2000-2010: A population-based retrospective cohort study. *Osteoporosis International* 2014;**25:** S170.

207. Duijnisveld BJ, Koenraadt KLM, van Steenbergen LN, Bolder SBT. Mortality and revision rate of cemented and uncemented hemiarthroplasty after hip fracture: an analysis of the Dutch Arthroplasty Register (LROI). *Acta Orthop* 2020;**91:** 408-413.

208. Ekinci O, Yanik S, Terzioʇlu B, Yilmaz Akyüz E, Dokuyucu A, Erdem S. The effect of calcium B-hydroxy-B-methylbutyrate, vitamin D and protein supplementation on postoperative immobilization in elderly malnourished patients with hip fracture: A randomized controlled study. *Clinical Nutrition* 2015;**34:** S102.

209. Flury A, Finsterwald M, Dimitriou D, Helmy N, Hasler J, Antoniadis A. Should advanced age be a contraindication to total hip arthroplasty in femoral neck fracture patients? A matched-control, retrospective study. *J Orthop* 2020;**17:** 25-29.

210. Guo J, Di J, Gao X, Zha J, Wang X, Wang Z*, et al.* Discriminative Ability for Adverse Outcomes After Hip Fracture Surgery: A Comparison of Three Commonly Used Comorbidity-Based Indices. *Gerontology* 2021**:** 1-13.

211. Koren-Hakim T, Otzrateni I, Weiss A, Grosman B, Frishman S, Beloosesky Y. Relationship between nutritional status of hip fracture operated elderly patients and functioning comorbidity and outcome. *Clinical Nutrition, Supplement* 2011;**6:** 15.

212. Koren-Hakim T, Weiss A, Hershkovitz A, Otzrateni I, Grosman B, Frishman S*, et al.* Comparison of the MNA-SF, NRS-2002 and must adequacy to assess malnutrition in hip fracture operated elderly patients. *Clinical Nutrition, Supplement* 2012;**7:** 179.

213. Makridis KG, Zourntou SE, Karachalios TS, Badras LS, Malizos KN. Prognostic factors in hip fractures: What is the ideal patient profile for an optimal treatment outcome. *Osteoporosis International* 2018;**29:** S160.

214. Malafarina V, Malafarina C, Biain Ugarte A, Martinez JA, Abete Goñi I, Zulet MA. Factors Associated with Sarcopenia and 7-Year Mortality in Very Old Patients with Hip Fracture Admitted to Rehabilitation Units: A Pragmatic Study. *Nutrients* 2019;**11**.

215. Malafarina V, Malafarina C, Ugarte AB, Martinez JA, Goñi IA, Zulet MA. Factors associated with sarcopenia and 7-year mortality in very old patients with hip fracture admitted to rehabilitation units: A pragmatic study. *Nutrients* 2019;**11**.

216. Malafarina V, Reginster JY, Cabrerizo S, Bruyère O, Kanis JA, Martinez JA*, et al.* Nutritional Status and Nutritional Treatment Are Related to Outcomes and Mortality in Older Adults with Hip Fracture. *Nutrients* 2018;**10**.

217. Quach LH, Jayamaha S, Whitehouse SL, Crawford R, Pulle CR, Bell JJ. Comparison of the Charlson Comorbidity Index with the ASA score for predicting 12-month mortality in acute hip fracture. *Injury* 2020;**51:** 1004-1010.

218. Rikkonen T, Poole K, Sirola J, Sund R, Honkanen R, Kröger H. Long-term effects of functional impairment on fracture risk and mortality in postmenopausal women. *Osteoporos Int* 2018;**29:** 2111-2120.

219. Schuijt HJ, Bos J, Smeeing DPJ, Geraghty O, van der Velde D. Predictors of 30-day mortality in orthogeriatric fracture patients aged 85 years or above admitted from the emergency department. *Eur J Trauma Emerg Surg* 2021;**47:** 817-823.

220. Chang CB, Yang RS, Chang LY, Peng JK, Tsai KS, Huang WJ*, et al.* Fracture types affect clinical outcomes of patients managed within the fracture liaison and osteoporosis medication management services. *Sci Rep* 2019;**9:** 10089.

221. Crijns TJ, Caton T, Teunis T, Davis JT, McWilliam-Ross K, Ring D*, et al.* Longer Length of Stay Increases 1-year Readmission Rate in Patients Undergoing Hip Fracture Surgery. *Arch Bone Jt Surg* 2018;**6:** 492-500.

222. Fernandez Tormos E, Limousin Aranzabal B, Arraiz Diaz C, Marín Peña O, Larrainzar Garijo R. Risk factors for postmenopausal osteoporosis do not characterize predictors for second hip fracture. *Osteoporosis International* 2016;**27:** S196.

223. Grigorie D, Sucaliuc A, Johansson H, Kanis JA, McCloskey E. Incidence of hip fracture in Romania and the development of a Romanian FRAX model. *Calcif Tissue Int* 2013;**92:** 429-436.

224. Jeschke E, Citak M, Günster C, Halder AM, Heller KD, Malzahn J*, et al.* Obesity Increases the Risk of Postoperative Complications and Revision Rates Following Primary Total Hip Arthroplasty: An Analysis of 131,576 Total Hip Arthroplasty Cases. *J Arthroplasty* 2018;**33:** 2287-2292.e2281.

225. Jutberger H, Ohlsson C, Lorentzon M, Kindblom J, Lerner U, Johansson H*, et al.* Elderly men with diabetes sustain hip fractures at higher BMD. Mr Os Sweden. *Bone* 2011;**48:** S70.

226. Kempegowda H, Richard R, Borade A, Tawari A, Graham J, Suk M*, et al.* Obesity Is Associated With High Perioperative Complications Among Surgically Treated Intertrochanteric Fracture of the Femur. *J Orthop Trauma* 2017;**31:** 352-357.

227. Lewis J, Estrada K, Uitterlinden A, Rivadeneira F, Prince R. Interaction of a single genotype and body mass index for prediction of hip fracture risk: A prospective cohort study. *Journal of Bone and Mineral Research* 2013;**28**.

228. Liu X, Dong Z, Li J, Feng Y, Cao G, Song X*, et al.* Factors affecting the incidence of surgical site infection after geriatric hip fracture surgery: a retrospective multicenter study. *J Orthop Surg Res* 2019;**14:** 382.

229. Malik AT, Quatman CE, Phieffer LS, Ly TV, Wiseman J, Khan SN. The impact of metabolic syndrome on 30-day outcomes in geriatric hip fracture surgeries. *European Journal of Orthopaedic Surgery and Traumatology* 2019;**29:** 427-433.

230. Monov S, Monova D, Ivanova M. Bone mineral density, fracture risk and associated factors in patients with hype-ruricemia. *Osteoporosis International* 2017;**28:** S245.

231. Myint MWW, Wu J, Wong E, Chan SP, To TSJ, Chau MWR*, et al.* Clinical benefits of oral nutritional supplementation for elderly hip fracture patients: A single blind randomised controlled trial. *Age Ageing* 2013;**42:** 39-45.

232. Papadimitriou N, Tsilidis KK, Orfanos P, Benetou V, Ntzani EE, Soerjomataram I*, et al.* Burden of hip fracture using disability-adjusted life-years: a pooled analysis of prospective cohorts in the CHANCES consortium. *Lancet Public Health* 2017;**2:** e239-e246.

233. Venuti D, Barisone M, Fenati C. Hip fracture and nutritional implications: Not for declining to bone! *Italian Journal of Medicine* 2014;**8:** 133.

234. Zhang JC, Matelski J, Gandhi R, Jackson T, Urbach D, Cram P. Can Patient Selection Explain the Obesity Paradox in Orthopaedic Hip Surgery? An Analysis of the ACS-NSQIP Registry. *Clin Orthop Relat Res* 2018;**476:** 964-973.

235. Walter S, Mackenbach J, Newson R, Hofman A, Tiemeier H. Obesity, incident disease, and mortality - The obesity paradox revisited. *American Journal of Epidemiology* 2011;**173:** S235.

236. Xing F, Luo R, Chen W, Zhou X. The risk-adjusted Charlson comorbidity index as a new predictor of one-year mortality rate in elderly Chinese patients who underwent hip fracture surgery. *Orthop Traumatol Surg Res* 2021;**107:** 102860.

237. Zajonz D, Brand A, Lycke C, Özkurtul O, Theopold J, Spiegl UJA*, et al.* Risk factors for early infection following hemiarthroplasty in elderly patients with a femoral neck fracture. *Eur J Trauma Emerg Surg* 2019;**45:** 207-212.

238. Fakhry SM, Morse JL, Garland JM, Wilson NY, Shen Y, Wyse RJ*, et al.* Increasing BMI is associated with higher mortality, worsening outcomes and highly specific injury patterns following trauma: A multi-institutional analysis of 191,274 patients. *J Trauma Acute Care Surg* 2021;**90:** 376-383.
