# Supplementary material for: Inverse relation of body weight with short-term and long-term mortality following hip fracture surgery: a meta-analysis
Source: J Orthop Surg Res. 2022 Apr 26;17:249. doi: 10.1186/s13018-022-03131-3 (PMC9044716; doi:10.1186/s13018-022-03131-3)
Supplement: Supplementary file 3 — Additional file 3. Quality assessment of the included studies on NOS-based methodological analysis. [file 13018_2022_3131_MOESM3_ESM.docx]

**Table S3**. The methodological quality of included studies. Studies were ranked using the Newcastle–Ottawa Scale (NOS).

|  | Flodin et al. (28) | Kimura et al. (29) | Modig et al. (12) | Müller et al. (27) | Pederson et al.(17) | Prieto-Alhambra  et al. (22) | Solbakken et al. (21) | Tahir et al. (18) | Akinleye et al. (24) | Kirkland et al. (26) | Chaudry et al. (25) |
| --- | --- | --- | --- | --- | --- | --- | --- | --- | --- | --- | --- |
| Representativeness of the exposed cohort | 0 | 0 | 0 | 0 | 1 | 1 | 1 | 0 | 1 | 0 | 1 |
| Selection of the non-exposed cohort | 1 | 1 | 1 | 1 | 1 | 1 | 1 | 1 | 1 | 1 | 1 |
| Ascertainment of exposure | 1 | 1 | 1 | 1 | 1 | 0 | 1 | 1 | 0 | 1 | 1 |
| Precision of Exposure Dose Ascertainment | 0 | 0 | 0 | 0 | 0 | 0 | 0 | 0 | 0 | 0 | 0 |
| Ascertainment of exposure done prospectively or retrospectively | 1 | 0 | 1 | 0 | 1 | 0 | 1 | 1 | 1 | 0 | 0 |
| Demonstration that outcome of interest was not present at start of study, OR baseline assessment | 1 | 0 | 0 | 0 | 0 | 0 | 1 | 1 | 0 | 0 | 0 |
| Adjustment for confounding (rendering comparability of cohorts on the basis of the design or analysis) | 0 | 0 | 0 | 0 | 0 | 0 | 1 | 0 | 1 | 0 | 0 |
| Assessment of outcome | 1 | 1 | 1 | 0 | 1 | 1 | 1 | 1 | 1 | 0 | 1 |
| Was follow-up long enough for outcomes to occur? | 1 | 1 | 1 | 1 | 1 | 1 | 1 | 1 | 1 | 1 | 1 |
| Adequacy of follow-up of cohorts | 1 | 1 | 0 | 1 | 1 | 1 | 1 | 0 | 0 | 1 | 1 |
| Total score | **7** | **5** | **5** | **5** | **7** | **5** | **9** | **6** | **6** | **5** | **6** |
