# Supplementary figures and images for: Inverse relation of body weight with short-term and long-term mortality following hip fracture surgery: a meta-analysis
Source: J Orthop Surg Res. 2022 Apr 26;17:249. doi: 10.1186/s13018-022-03131-3 (PMC9044716; doi:10.1186/s13018-022-03131-3)

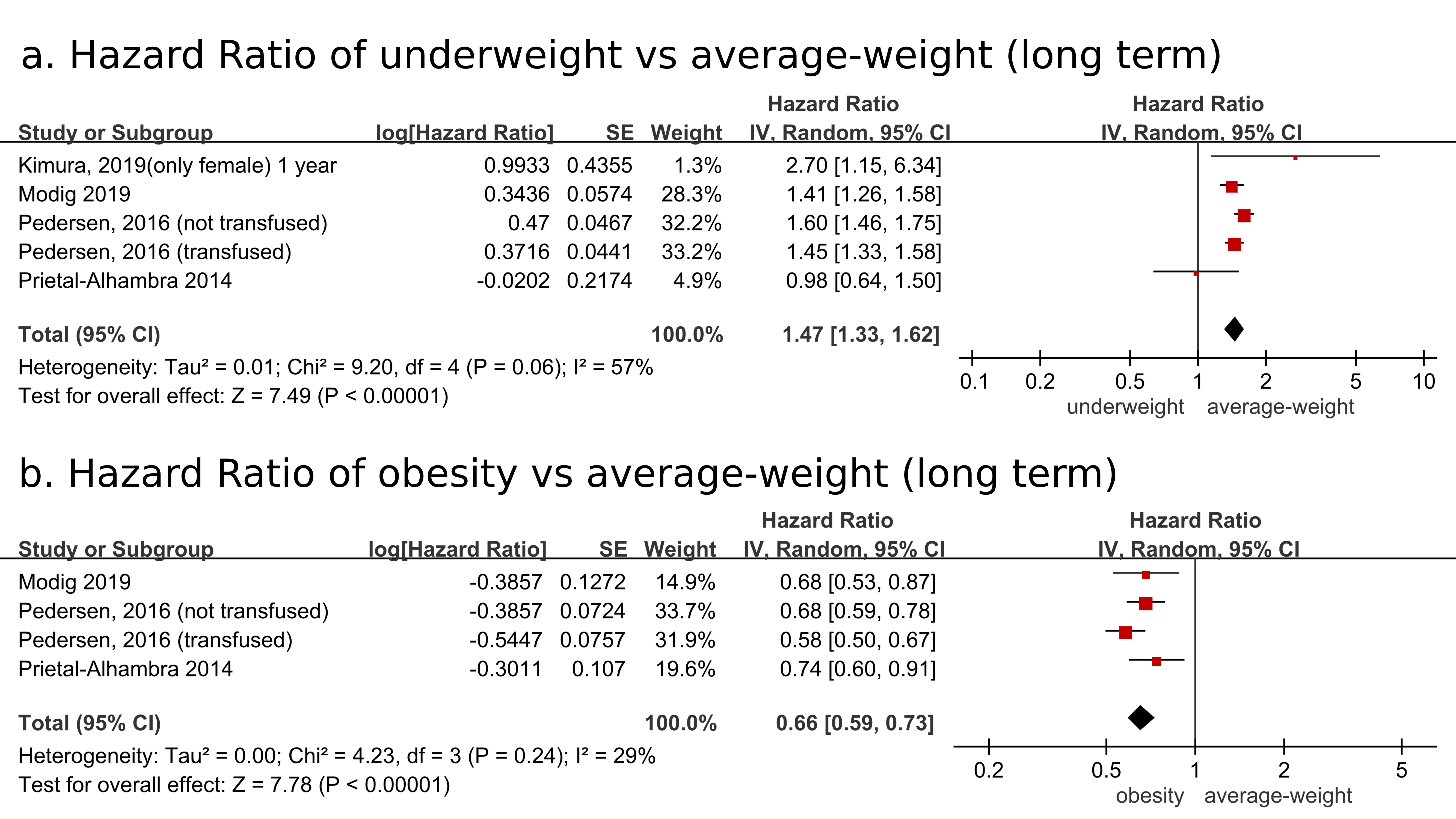

Supplement: Supplementary file 4 — Additional file 4. Comparison of long-term mortality rate in hazard ratios after hip fracture surgery (a) underweight vs average-weight groups (b) obesity vs average weight group. [file 13018_2022_3131_MOESM4_ESM.tiff]
